# Antidepressants and Breast and Ovarian Cancer Risk: A Review of the Literature and Researchers' Financial Associations with Industry

**DOI:** 10.1371/journal.pone.0018210

**Published:** 2011-04-06

**Authors:** Lisa Cosgrove, Ling Shi, David E. Creasey, Maria Anaya-McKivergan, Jessica A. Myers, Krista F. Huybrechts

**Affiliations:** 1 The Edmond J. Safra Center for Ethics, Harvard University, Cambridge, Massachusetts, United States of America; 2 Department of Nursing and Health Sciences, University of Massachusetts, Boston, Massachusetts, United States of America; 3 Department of Counseling and School Psychology, University of Massachusetts, Boston, Massachusetts, United States of America; 4 Department of Psychiatry, Harvard Medical School, Boston, Massachusetts, United States of America; 5 College of Social Sciences, University of Phoenix, Yuma, Arizona, United States of America; 6 Division of Pharmacoepiodemiology and Pharmacoeconomics, Department of Medicine, Brigham and Women's Hospital and Harvard Medical School, Boston, Massachusetts, United States of America; Virginia Commonwealth University, United States of America

## Abstract

**Background:**

Antidepressant (AD) use has been purported to increase the risk of breast and ovarian cancer, although both epidemiological and pre-clinical studies have reported mixed results [1]–[6]. Previous studies in a variety of biomedical fields have found that financial ties to drug companies are associated with favorable study conclusions [7].

**Methods and Findings:**

We searched English-language articles in MEDLINE, PsychINFO, the Science Citations Index and the Cochrane Central Register of Controlled Clinical Trials (through November 2010). A total of 61 articles that assessed the relationship between breast and ovarian cancer and AD use and articles that examined the effect of ADs on cell growth were included. Multi-modal screening techniques were used to investigate researchers' financial ties with industry. A random effects meta-analysis was used to pool the findings from the epidemiological literature. Thirty-three percent (20/61) of the studies reported a positive association between ADs and cancer. Sixty-seven percent (41/61) of the studies reported no association or antiproliferative effect. The pooled odds ratio for the association between AD use and breast/ovarian cancer in the epidemiologic studies was 1.11 (95% CI, 1.03–1.20). Researchers with industry affiliations were significantly less likely than researchers without those ties to conclude that ADs increase the risk of breast or ovarian cancer. (0/15 [0%] vs 20/46 [43.5%] (Fisher's Exact test P = 0.0012).

**Conclusions:**

Both the pre-clinical and clinical data are mixed in terms of showing an association between AD use and breast and ovarian cancer. The possibility that ADs may exhibit a bi-phasic effect, whereby short-term use and/or low dose antidepressants may increase the risk of breast and ovarian cancer, warrants further investigation. Industry affiliations were significantly associated with negative conclusions regarding cancer risk. The findings have implications in light of the 2009 USPSTF guidelines for breast cancer screening and for the informed consent process.

## Introduction

There is some evidence that antidepressant (AD) use is associated with increased cancer risk [1]–[6], although the results from both epidemiological and pre-clinical studies have been mixed. Reviewing the evidence is a critical public health issue in light of the increasing prevalence of AD use, especially among women, and in light of the fact that 1 in 8 women will be diagnosed with cancer of the breast during their lifetime [8]. Ovarian cancer is the second most frequently occurring female reproductive cancer and causes more deaths than any other gynecological cancer [9]. In the US alone, over 27 million people are taking an AD [10], most of whom are women, for they are twice as likely as men to be diagnosed with Major Depressive Disorder and up to three times more likely to be diagnosed with Dysthymic Disorder [11]. ADs are increasingly being prescribed for other conditions such as hot flashes, headache, back pain, neuropathy, sleep-related conditions, anxiety spectrum disorders, eating disorders, and fibromyalgia [12].

Experimental studies have demonstrated that some ADs promote tumor growth in animals [5], [6], [13] although the exact mechanism by which antidepressants may increase the risk of tumors is currently unknown. Some ADs, especially SSRIs, are potent inhibitors of the cytochrome P450 monooxygenase enzymatic system (a system that metabolizes antineoplastic as well as other agents) [14]. The expanding pre-clinical and clinical research on CYP450 enzymes and the deleterious effects of these enzymes on the metabolism and therapeutic efficacy of tamoxifen and other antineoplastic agents [15] has led to concerns that ADs may directly enhance tumor cell proliferation.

The results of epidemiological studies examining an association between AD use and breast cancer [1]–[4] have been inconsistent. Several of these studies relied on older datasets from before the widespread use of SSRIs [16]–[18]. Given the number of women who are currently taking SSRIs, it is important to systematically review the most recent pre-clinical and epidemiological studies that have included this class of antidepressants. Including both epidemiological and pre-clinical studies allows for a more thorough assessment of the literature regarding breast and ovarian cancer risk and AD use by addressing the issue of biological plausibility.

A growing area of concern in the biomedical sciences, including oncology, is the potential for financial conflict of interest to influence the outcome of a study [19], [20]. The specific mechanisms by which industry influence may operate remain unclear and are most likely varied. For example, researchers who had financial associations with manufacturers of drugs have been found to be less likely to criticize the safety of the drug than researchers without those ties [21]. Other studies have shown an association between company sponsorship and favorable study outcome [22]. To our knowledge, our study is the first one to examine researchers' industry ties and their conclusions on the relationship between breast and ovarian cancer risk and AD use.

## Methods

### Data Sources

A list of FDA approved antidepressants was generated and a search was conducted for English language articles in MEDLINE (via ISI Web of Knowledge), PSYCINFO, Science Citations Index, and the Cochrane Database of Controlled Trials Register (no year limits through November 2010). Following Lawlor et al. [23], various Medical Subject Headings (MeSH) and free text terms were combined (e.g., ovarian cancer and antidepressants; breast carcinoma SSRIs, Tricyclics, MAO inhibitors”) in order to generate a list of published human and animal studies that examined the relationship between ADs and breast and ovarian cancer. (See Text S1 for a detailed description of the search strategy.) We identified additional studies by searching the reference section of selected articles. One of the authors (DC) has expertise in psychopharmacology and psychogenomics and provided titles and abstracts of additional studies.

### Study Selection

The main criteria for study selection were 1) clinical studies (including randomized controlled trials, case-control, prospective and retrospective cohort studies) that assessed the relationship between AD use and breast and ovarian cancer and 2) preclinical studies that that assessed the carcinogenic properties of ADs on tumor lines or in animals. Carcinogenic was defined as “mutagenetic,” “clastogenetic,” “epigenetic,” “mitogenetic,” “cytogenetic,” “genotoxic,” “tumorigenic,” neoplastic, “anti-apoptotic,” “teratogenetic,” “associated with sister chromatid exchanges,” and “induction of genomic instability.” We excluded studies that examined medications other than ADs (e.g., antihistamines which have a similar molecular structure to ADs), and human studies that did not empirically assess the relationship between ADs and breast and ovarian cancer (e.g., anecdotal case reports). We also excluded epidemiological studies that assessed the relationship between AD use and other cancer types (e.g. lung) as they were beyond the scope of this review.

**Figure 1:**
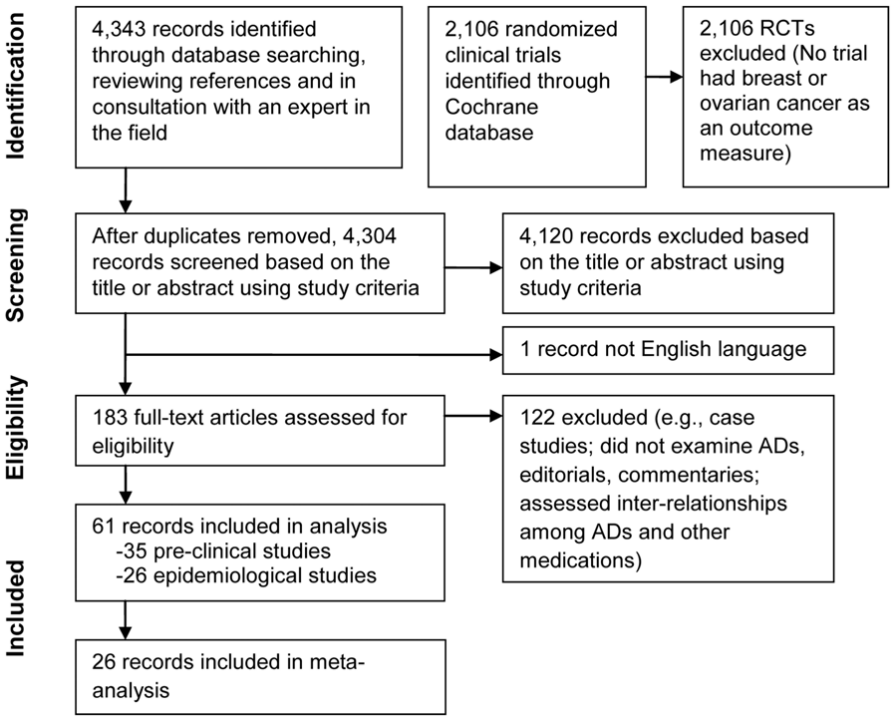
Literature search and study selection.

Electronic database searches, reference mining and consultation with an expert in the field yielded 4,343 citations. Although our search identified 2,106 clinical trials in the Cochrane Central Register of Controlled Trials, no randomized or quasi randomized controlled trials were found to have breast or ovarian cancer as any outcome measure. After removing duplicates, 4,120 were excluded based on the title or abstract review (using the study selection criteria described above), 184 records were requested and 183 (99.5%) were retrieved and reviewed. Of the retrieved records, 122 were rejected because they did not meet study inclusion criteria (e.g., case reports, editorials, commentaries, assessed medications other than ADs). Studies that assessed the inter-relationship among serotonin, SSRIs, certain TCAs, prolactin, and tamoxifen, and how these inter-relationships affect pharmacodynamics and cancer risk, were retrieved and underwent a preliminary review. The remaining articles (N = 61) underwent full review (Figure 1).

### Data Extraction

Any concerns regarding eligibility were discussed and agreed upon by the investigators. For the 61 articles that met our study criteria, one investigator (LC) originally abstracted data about the relationship between ADs and cancer and another (LS for the epidemiological studies, DC for the pre-clinical ones) independently reviewed the data. Abstraction of the data took place prior to screening for industry associations and reviewers were blinded with regard to knowledge about the researchers' industry associations. Because a main objective of the present study was to identify the original researchers' stated conclusions regarding breast and ovarian cancer risk and ADs, (i.e., No Association vs. Yes Association) there was little ambiguity in terms of how to abstract the data. There was one discrepancy which was resolved by consultation with an outside expert who was blinded to knowledge about industry associations.

### Data Synthesis

Twenty–six epidemiological studies were reviewed [1], [2], [4], [16]–[18], [24]–[43] and designated according to authors' findings as A) No association B) Yes Association. The latter category included those studies that found subsamples of women with elevated risks (e.g., women over 50) or researchers who reported one or more statistically significant associations between AD use and cancer (e.g., significant findings for SSRIs on ER−PR−, ER+/PR− tumors). In addition, we performed a meta-analysis using the main effect estimate from each of the 26 epidemiological studies. Most studies reported effect estimates as odds ratios. In all cases where a relative risk or hazard ratio was reported, outcome was rare, and we assumed that these measures approximate the odds ratio. In studies with two main effects (one for SSRI exposure and one for TCA exposure or one for risk of breast cancer and one for risk of ovarian cancer), we used the estimate for the SSRI exposure or for risk of breast cancer, since the effect of SSRI exposure on the risk of breast cancer was the most commonly investigated causal effect. As a sensitivity analysis, we calculated pooled estimates with the opposite set of estimates included (including the estimate for TCA exposure from studies that reported separate estimates for SSRI and TCA use and including the estimate for ovarian cancer from studies that reported separate estimates for breast and ovarian cancer). Analysis was also performed separately in the subset of studies that reported investigating SSRI exposure and the subset of studies that reported investigating TCA exposure. A random-effects meta-analysis model was used to account for the heterogeneity across studies [44] and a funnel plot was examined for evidence of publication bias. Table S1 provides a detailed review and summarizes the study design, participants, exposure definitions, outcomes and conclusions of these 26 epidemiological studies (see also Table S2).

Thirty-five pre-clinical (i.e., animal and laboratory) studies were reviewed [5], [6], [13], [45]–[76] and designated according to researchers' findings regarding ADs as being carcinogenic. Following this review the articles were then designated as A) Positive for carcinogenesis or B) Negative for carcinogenesis. The “Negative” designation also included studies that found ADs to have an antineoplastic/antiproliferative effect on cells or tumors. A detailed review of the pre-clinical studies is provided (see Table S3; see also Table S4).

We were also interested in discerning whether there was a connection between industry ties and qualitative conclusions about cancer risk and AD use. Using multimodal screening techniques, we examined the relationship between principal investigators' conclusions and their financial ties to the pharmaceutical industry. The multi-modal screening methods applied included: MEDLINE, (used to identify published papers that disclose author financial interests), and the following internet search engines: Lexis-Nexis, the U.S. Patent and Trademark Office internet site on patents awarded, and the internet site for SEC filings. The development of both criteria for “holding a financial interest” and for parameters regarding the timing of financial associations (5 years before and five years after a study was published) were based on standards set by government bodies, journals, and professional societies as well as based on prior publications [77], . Financial associations for this study included: being an employee of a pharmaceutical company; receiving honorarium; receiving research funding or research materials (equipment, drugs, cell cultures, etc); holding equity in a company; being a member or serving as a consultant to a pharmaceutical corporate board; providing expert testimony on behalf of the pharmaceutical company; and holding a patent, patent application or royalties on AD medication. Two investigators (LC and MM) screened for financial associations and two (DC and LS) reviewed the disclosures. No author was coded as having a financial connection unless there was unambiguous information confirming the relationship. Only affiliations with manufacturers of ADs were included in our analysis. Only PIs were screened for industry associations, thereby minimizing the possibility that studies with multiple authors would be more likely to have an author with an industry association (vs. single authored studies). The statistical relationship between qualitative conclusions and funding source was analyzed using Fisher's Exact test. Statistical analysis was performed with Stata version 8.0 (Stata Corp, College Station, TX, USA).

## Results

**Figure 2:**
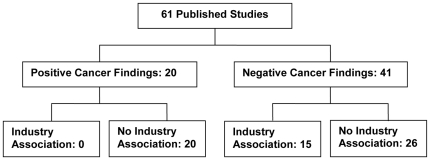
Summary of results.

**Figure 3:**
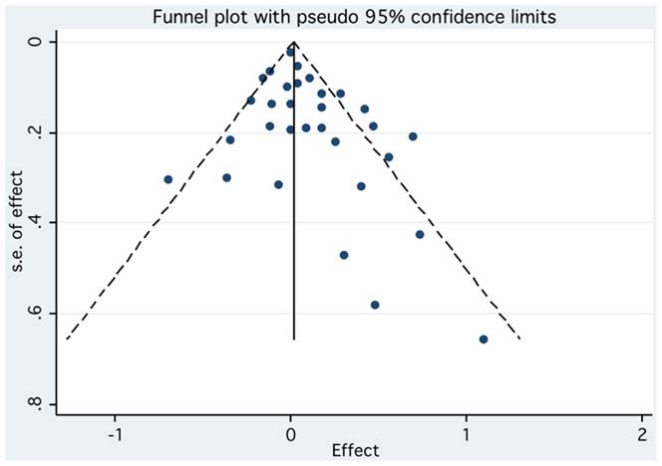
Funnel plot evaluating the presence of publication bias.

Over one-third (38.4% or 10/26) of the epidemiological studies reported statistically significant findings, of which 8 were case-control studies and 2 cohort studies. Almost one-third (28.6% or 10/35) of the pre-clinical studies reported a positive finding with regard to the AD agent being carcinogenic, a tumor promoter, or genotoxic. Thus, 32.8% (20/61) of the epidemiological and pre-clinical studies reported an association between ADs and cancer (Figure 2). There was no strong evidence of publication bias for the epidemiological studies based on a visual inspection of the funnel plot, although there is some asymmetry suggesting smaller studies with negative associations might be under-represented in the literature (Figure 3).

**Figure 4:**
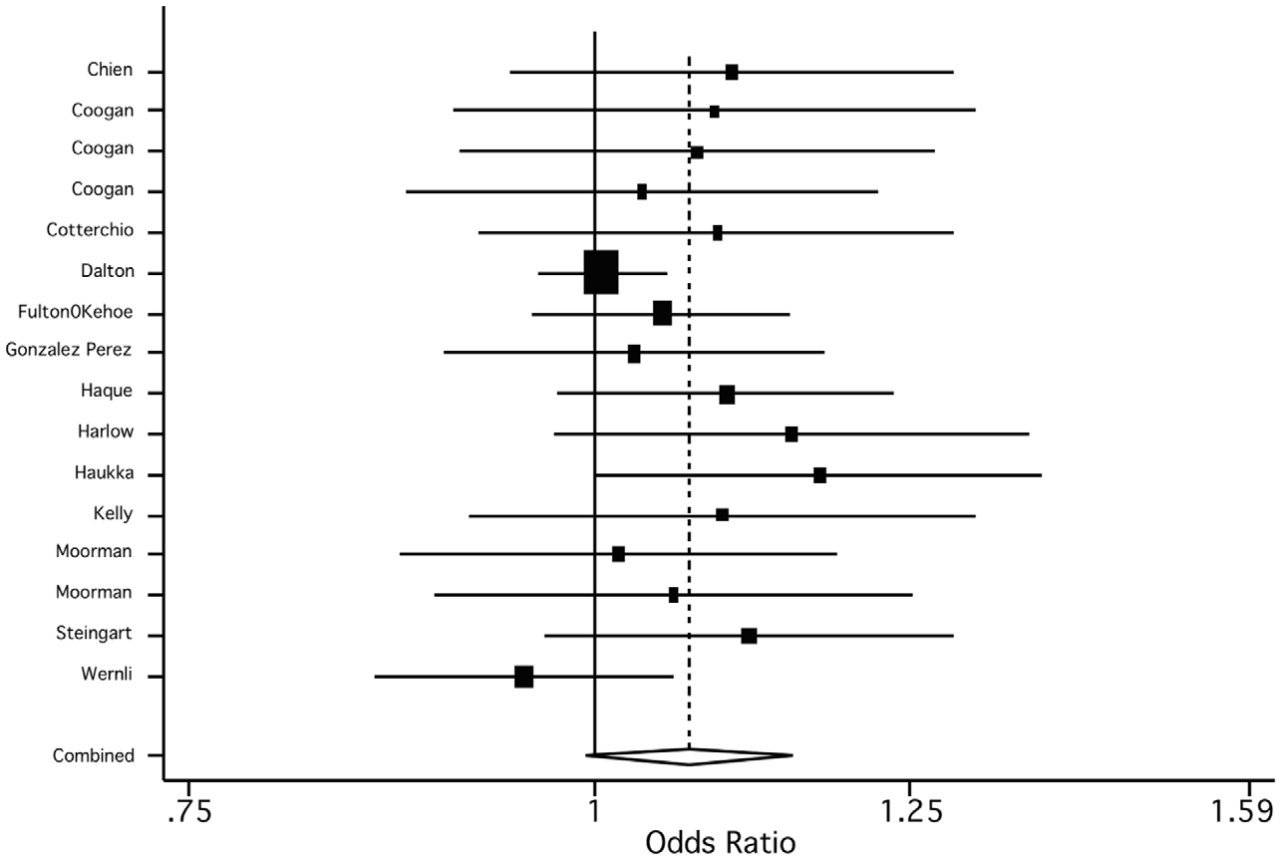
Empirical Bayes estimates of the study specific and the pooled estimate for SSRIs.

**Figure 5:**
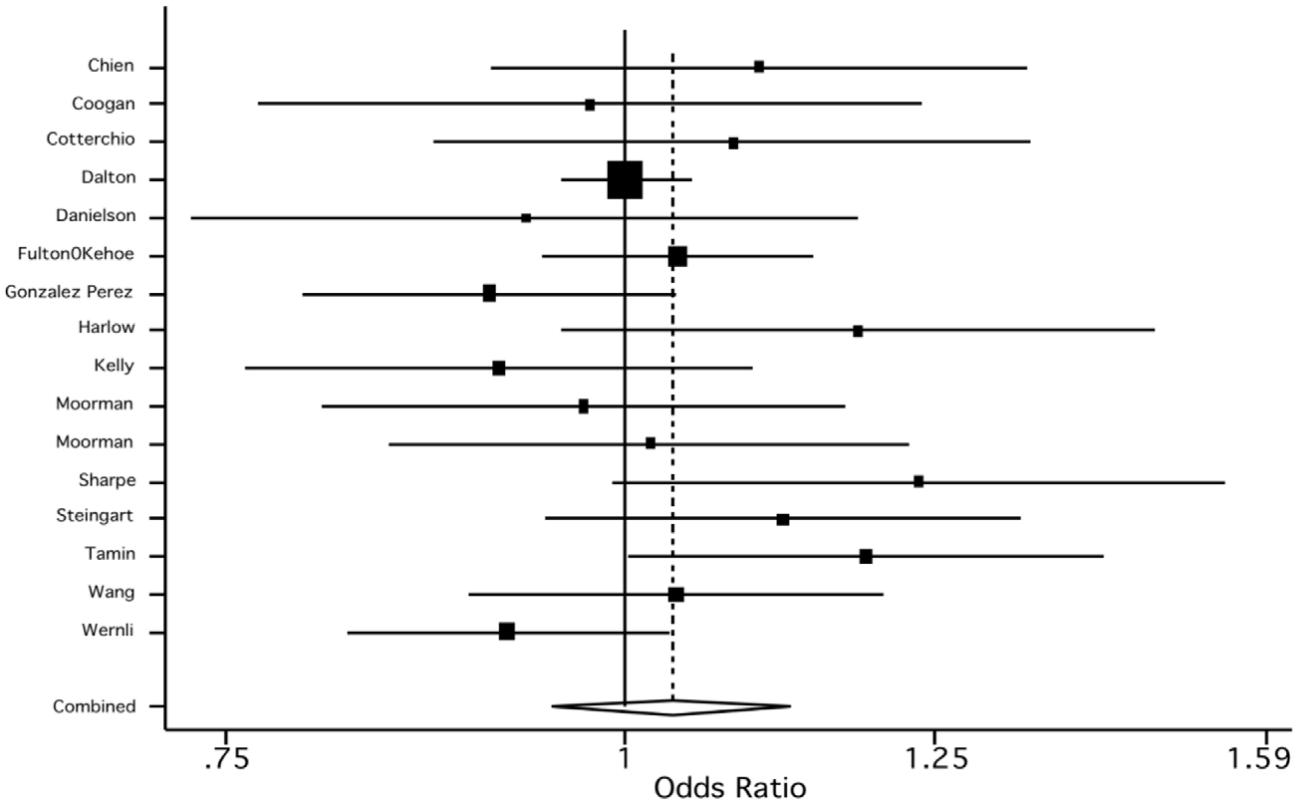
Empirical Bayes estimates of the study specific and the pooled estimate for TCAs.

The pooled odds ratio of 1.11 (95% CI, 1.03–1.20), suggests that ADs may be associated with a small increase in breast and ovarian cancer risk (see Table 1). When we instead included the estimates for TCA and ovarian cancer, the effect was attenuated (1.05, 95% CI, 0.98–1.14). When we estimated the pooled effect separately by AD class, we found a slightly stronger effect for SSRIs (1.07, 95% CI, 0.99–1.51) than for TCAs (1.04, 95% CI 0.95–1.13). Moreover, all studies except one included in the SSRI analysis reported a positive association (Figure 4), whereas the evidence for TCAs was more mixed, with 9 studies reporting a positive association, 6 studies a negative association, and one study a null association (Figure 5).

**Table 1:** Empirical Bayes effect estimates and weights for the 26 epidemiological studies included in meta-analysis.

Study	Weight	Effect Estimate	95% CI	
Chien	26.08	1.15	0.94	1.40
Coogan	2.23	1.15	0.89	1.49
Coogan	18.60	1.11	0.89	1.38
Coogan	19.33	1.03	0.83	1.28
Cotterchio	18.60	1.14	0.91	1.41
Dalton	57.52	1.00	0.96	1.05
Danielson	9.10	0.98	0.77	1.25
Davis	15.27	1.16	0.92	1.45
Dublin	15.66	0.99	0.79	1.24
Fulton Kehoe	50.83	1.05	0.95	1.16
Gonzalez Perez	37.84	1.03	0.88	1.20
Haque	43.40	1.12	0.98	1.28
Harlow & Cramer	5.02	1.17	0.91	1.51
Harlow	19.16	1.25	1.01	1.55
Haukka	25.51	1.27	1.05	1.55
Kato	12.22	1.22	0.96	1.54
Kelly	8.41	1.16	0.91	1.48
Moorman	28.01	1.01	0.83	1.22
Moorman	18.32	1.07	0.86	1.34
Sharpe	16.54	1.31	1.05	1.64
Steingart	33.17	1.16	0.98	1.38
Tamim	33.29	1.23	1.04	1.46
Wallace	2.81	1.13	0.87	1.46
Wang	39.46	1.06	0.92	1.23
Weiss	8.51	1.08	0.85	1.38
Wernli	47.30	0.93	0.83	1.05

None of the 4 epidemiological studies for which the principal investigator (PI) had industry ties reported a positive association between AD use and cancer risk. Of the 22 studies for which the PI had no industry ties, 45% (10/22) reported positive findings. Likewise, none of the 11 pre-clinical studies for which the PI had industry ties reported positive cancer findings. Of the 24 studies for which the PI had no industry ties, 42% (10/24) reported that ADs were carcinogenic, tumor promoters, genotoxic, or neoplastic. Thus, in total, none of the 15 researchers who had industry ties reported positive cancer findings, compared with 43% of the researchers without industry ties. There was a statistically significant relationship between researchers' industry ties and conclusions regarding ADs and cancer (2-sided Fisher's Exact test P = 0.0012).

## Discussion

The meta-analysis of epidemiological studies suggests there may be a modest increase in the risk of breast/ovarian cancer with the use of ADs, especially SSRIs. Other potential explanatory factors for this finding include the role of depression itself rather than treatment with AD [79], failure to distinguish between short- and long-term exposure, and a failure to examine a possible non-linear dose related response as the mechanism for malignant cell proliferation [5], [76], [80], [81]. This bi-phasic phenomenon is characterized by “low-dose stimulation and high-dose inhibition” [76] of malignant cell proliferation. Thus, rather than having fewer and less severe side effects, short-term use and/or low dose antidepressants could increase the risk of breast and ovarian cancer in women or exacerbate cancer cell growth in women in the early stages of breast and ovarian cancer. Large scale prospective cohort studies of women using SSRIs are needed in order to determine if ADs cause and/or enhance breast and ovarian tumor growth. Specifying induction time and exposure also will allow future researchers to more accurately assess for dose and duration effects.

The fact that industry affiliations were significantly associated with negative findings regarding cancer risk raises public health and policy issues because there is increasing evidence that financial ties among industry, investigators, and academic institutions can affect the research process [82]–[84]. For example, re-evaluations of liver tissue of rats exposed to dioxin resulted in different conclusions about the liver cancer rates in those rats; industry sponsored reevaluations were associated with fewer cancer characterizations [85]–[87], which had an effect on the policy recommendations. Thus, financial associations between biomedical researchers and the pharmaceutical industry may result in the publication of incomplete or inaccurate results and imbalanced recommendations [20], [88], [90]. This is not to suggest that researchers with financial associations to industry intentionally designed studies to produce results favorable to manufacturers of ADs or that they misrepresented their results. The existence of researchers' industry associations points to a *generic* risk [91] that a financial conflict of interest may compromise the research process or undermine public trust; “conflict[s] of interest do not imply that any [specific researcher] is improperly motivated.” As Thompson, 2009, astutely notes, it is virtually impossible to determine that a particular decision during the research process was motivated by secondary interests (e.g., financial gain). Although space precludes a more extensive discussion, we agree with Thompson [91] that the generic risk incurred by financial conflicts of interest undermines public trust. “[T]he point is to minimize or eliminate circumstances that would cause reasonable persons to suspect that professional judgment has been improperly influenced, *whether or not it has* (emphasis added)” [91, p137]. Thus, we need to develop mechanisms and policies that enhance public trust in the biomedical field (e.g., by creating ‘firewalls’ between industry and academic researchers).

Moreover, there are many posited mechanisms for the link between conclusions and industry ties—mechanisms that can be subtle and unintentional. Our current regulatory system does not incentivize industry to develop maximally informative study designs [92], which may in turn reinforce implicit bias. For example, industry affiliated researchers are incentivized to design pre-clinical studies that use only those dosages that have been associated with antiproliferative effects. Our findings suggest that there is a need to either incentivize industry to use non-linear dose response models or change carcinogenicity guidelines (outlined below).

Given the lack of epidemiological data on low-dose SSRI use, the issue of informed consent becomes a complex and critical one. It is likely that women, especially women being prescribed adjunctive AD therapy for depression secondary to a breast or ovarian cancer diagnosis or as adjunctive therapy for pain and hot flashes, would want to know about the positive cancer findings of animal and human studies, the genotoxic properties of some ADs, as well as the potential neoplastic/antiproliferative findings reported in some studies. Additionally, our findings have implications in light of the latest U.S. Preventive Services Task Force (USPSTF) guidelines recommending that women between the ages of 40–49 do not need routine screening for breast cancer [93]. Non-high risk women between the ages of 40 and 49 who have taken ADs, especially low dose SSRIs, may want to continue to get yearly mammograms. Also, although some regulatory agencies suggest that researchers consider using middle and low doses for rodent carcinogenicity studies (e.g., the FDA), we recommend that agencies require researchers to use non-linear dose response models when assessing the carcinogenicity of drugs.

Our study has several limitations. First, some of the articles retrieved were published prior to the development of COI policies and standard disclosure practices. It is likely that our screening techniques missed some industry ties and disclosure depends upon honest reporting of those ties. Thus, our findings should be considered *de-minimis* figures. Second, although we searched multiple databases, we did not search EMBASE. However, in light of the documented retrieval congruence between MEDLINE and EMBASE [94] and the fact that we hand-reviewed the reference sections of most of the articles that met study criteria, it is unlikely that we would have missed a significant number of articles that would have changed our study results. However, future researchers should include EMBASE to ensure as complete a search as possible. Third, although a random-effects meta-analysis was conducted, results should be interpreted with caution given that the studies included were very heterogeneous in terms of – amongst others – design, patient population, outcomes measured, and follow-up times. Moreover, too few studies were available to conduct meaningful subgroup analyses accounting for some of these characteristics. Future research should include a systematic review of all epidemiological data on cancer risk and AD use. Progress in the field of pharmacogenomics has led to increasing concerns about the complex relationships among serotonin, SSRIs, certain TCAs, prolactin, and tamoxifen, and how these inter-relationships affect pharmacodynamics and cancer risk [95]. It is recommended that future research examine this body of literature and investigate the association between industry funding and qualitative conclusions regarding cancer risk.

## Supporting Information

Text S1 Search strategy.
(DOC)

Table S1 Summary of epidemiological studies on antidepressants and cancer risk.
(DOC)

Table S2 Results and study design of epidemiological studies.
(DOC)

Table S3 Summary of pre-clinical studies on antidepressants and cancer risk.
(DOC)

Table S4 Results regarding carcinogenicity from pre-clinical studies.
(DOC)

## References

[1] Cotterchio M, Kreiger N, Darlington G, Steingart A (2000). Antidepressant medication and breast cancer risk.. Am J Epidemiol.

[2] Kelly JP, Rosenberg L, Rao RS, Palmer JR, Shapiro S (1998). Is use of antidepressants associated with the occurrence of breast cancer?. Am J Epidemiol.

[3] Steingart AB, Cotterchio M (1995). Do antidepressants cause, promote, or inhibit cancers?. J Clin Epidemiol.

[4] Harlow BL, Cramer DW (1995). Self-reported use of antidepressants or benzodiazepine tranquilizers and risk of epithelial ovarian cancer: evidence from two combined case-control studies.. Cancer Causes Control.

[5] Brandes LJ, Arron RJ, Bogdanovic RP, Tong J, Zaborniak CLF (1992). Stimulation of malignant growth in rodents by antidepressant drugs at clinically relevant doses.. Cancer Res.

[6] Iishi H, Tatsuta M, Miyako B, Taniguchi H (1993). Enhancement by the tricyclic antidepressant, desipramine, of experimental carcinogenesis in rat colon induced by azoxymethane.. Carcinogenesis.

[7] Yank V, Rennie D, Bero LA (2007). Financial ties and concordance between results and conclusions in meta-analyses: retrospective cohort study.. BMJ.

[8] National Cancer Institute Surveillance and Epidemiology and End Results.. http://seer.cancer.gov/statfacts/html/breast.html.

[9] American Cancer Society.. http://www.cancer.org/downloads/STT/2008CAFFfinalsecured.pdf.

[10] Olfson M, Marcus SC (2009). National patterns in antidepressant medication treatment.. Arch Gen Psychiatry.

[11] American Psychiatric Association (2000). Diagnostic and statistical manual of mental disorders: DSM-IV-TR. 4th ed. Text rev.

[12] Roberts H (2007). Managing the menopause.. BMJ.

[13] Hilakivi-Clarke L, Wright A, Lippman ME (1993). DMBA-induced mammary tumor growth in rats exhibiting increased or decreased ability to cope with stress due to early postnatal handling or antidepressant treatment.. Physiol Behav.

[14] Spina E, Santoro V, D'Arrigo C (2008). Clinically relevant pharmacokinetic drug interactions with second-generation antidepressants: an update.. Clin Ther.

[15] Aubert RE, Stanek EJ, Yao J, Teagarden JR, Subar M (2009). Risk of breast cancer recurrence in women initiating tamoxifen with CYP2D6 inhibitors.. J Clin Oncol.

[16] Danielson DA, Jick H, Hunter JR, Stergachis A, Madsen S (1982). Nonestrogenic drugs and breast cancer.. Am J Epidemiol.

[17] Wang PS, Walker AM, Tsuang MT, Orav EJ, Levin R (2001). Antidepressant use and the risk of breast cancer: a non-association.. J Clin Epidemiol.

[18] Dublin S, Rossing MA, Heckbert SR, Goff BA, Weiss NA (2002). Risk of epithelial ovarian cancer in relation to use of antidepressants, benzodiazepines, and other centrally acting medications.. Cancer Causes Control.

[19] Friedberg M, Saffran B, Stinson TJ, Nelson W, Bennett CL (1999). Evaluation of conflict of interest in economic analyses of new drugs used in oncology.. JAMA.

[20] Angell M (2004). The truth about drug companies: how they deceive us and what to do about it.

[21] Stelfox HT, Chua G, O'Rourke K, Detsky AS (1998). Conflict of interest in the debate over calcium-channel antagonists.. N Engl J Med.

[22] Heres S, Davis J, Maino K, Jetzinger E, Kissling W (2006). Why olanzapine beats risperidone, risperidone beats quetiapine, and quetiapine beats olanzapine: an exploratory analysis of head-to-head comparison studies of second-generation antipsychotics.. Am J Psychiatry.

[23] Lawlor DA, Jüni P, Ebrahim E, Egger M (2003). Systematic review of the epidemiologic and trial evidence of an association between antidepressant medication and breast cancer.. J Clin Epidemiol.

[24] Steingart A, Cotterchio M, Kreiger N, Sloan M (2003). Antidepressant medication use and breast cancer risk: a case-control study.. Int J Epidemiol.

[25] Chien C, Li CI, Heckbert SR, Malone KE, Boudreau DM (2006). Antidepressant use and breast cancer risk.. Breast Cancer Res Treat.

[26] Coogan PF, Rosenberg L, Palmer JR, Strom BL, Stolley PD (2000). Risk of ovarian cancer according to use of antidepressants, phenothiazines, and benzodiazepines.. Cancer Causes Control.

[27] Coogan PF, Palmer JR, Strom BL, Rosenberg L (2005). Use of selective serotonin reuptake inhibitors and the risk of breast cancer.. Am J Epidemiol.

[28] Coogan PF, Strom BL, Rosenberg L (2008). SSRI use and breast cancer risk by hormone receptor status.. Breast Cancer Res Treat.

[29] Dalton SO, Johansen C, Mellemkjaer L, Sorensen HT, McLaughlin JK (2000). Antidepressant medications and risk for cancer.. Epidemiology.

[30] González-Pérez A, García Rodríguez LA (2005). Breast cancer risk among users of antidepressant medications.. Epidemiology.

[31] Haque R, Enger SM, Chen W, Petitti DB (2005). Breast cancer risk in a large cohort of female antidepressant medication users.. Cancer Lett.

[32] Harlow BL, Cramer DW, Baron JA, Titus-Ernstoff L, Greenberg ER (1998). Psychotropic medication use and risk of epithelial ovarian cancer.. Cancer Epidemiol Biomarkers Prev.

[33] Kato I, Zeleniuch-Jacquotte A, Toniolo PG, Akhmedkhanov A, Koenig K (2000). Psychotropic medication use and risk of hormone related cancers: the New York University Women's Health Study.. J Pub Health Med.

[34] Moorman PG, Grubber JM, Millikan RC, Newman B (2003). Antidepressant medications and their association with invasive breast cancer and carcinoma in situ of the breast.. Epidemiology.

[35] Moorman PG, Berchuck A, Calingaert B, Halabi S, Schildkraut JM (2005). Antidepressant medication use and risk of ovarian cancer.. Obstet Gynecol.

[36] Sharpe CR, Collet J-P, Belzile E, Hanley JA, Boivin J-F (2002). The effects of tricyclic antidepressants on breast cancer risk.. Br J Cancer.

[37] Tamim H, Boivin J-F, Hanley J, Stang MR, Collet J-P (2006). Risk of breast cancer in association with exposure to two different groups of tricyclic antidepressants.. Pharmacoepidemiol Drug Saf.

[38] Wallace RB, Sherman BM, Bean JA (1982). A case-control study of breast cancer and psychotropic drug use.. Oncology.

[39] Weiss SR, McFarland BH, Burkhart GA, Ho PT (1998). Cancer recurrences and secondary primary cancers after use of antihistamines or antidepressants.. Clin Pharmacol Ther.

[40] Wernli KJ, Hampton JM, Trentham-Dietz A, Newcomb PA (2009). Antidepressant medication use and breast cancer risk.. Pharmacoepidemiol Drug Saf.

[41] Haukka J, Sankila R, Klaukka T, Lonnqvist J, Niskanen L (2010). Incidence of cancer and antidepressant medication: record linkage study.. Int J Cancer.

[42] Davis S, Mirick DK (2007). Medication use and the risk of breast cancer.. Eur J Epidemiol.

[43] Fulton-Kehoe D, Rossing MA, Rutter C, Mandelson MT, Weiss NS (2006). Use of antidepressant medications in relation to the incidence of breast cancer.. Br J Cancer.

[44] DerSimonian R, Laird N (1986). Meta-analysis in clinical trials.. Control Clin Trials.

[45] Abdul M, Logothetis CJ, Hoosein NM (1995). Growth-inhibitory effects of serotonin uptake inhibitors on human prostate carcinoma cell lines.. J Urol.

[46] Basso AM, Depiante-Depaoli M, Molina VA (1992). Chronic variable stress facilitates tumoral growth: reversal by imipramine administration.. Life Sci.

[47] Bendele RA, Adams ER, Hoffman WP, Gries CL, Morton DM (1992). Carcinogenicity studies of fluoxetine hydrochloride in rats and mice.. Cancer Res.

[48] Brambilla G, Cavanna M, Faggin P, Pino A, Robbiano L (1982). Genotoxic activity of five antidepressant hydrazines in a battery of in vivo and in vitro short-term tests.. J Toxicol Environ Health.

[49] Clayson DB, Biancifiori C, Milia U, Santilli FEG, Severi L (1965). The induction of pulmonary tumors in BALB/c/Cb/Se mice by derivatives of hydrazine.. Lung tumours in animals: Proceedings of the third quadrennial conference on cancer.

[50] Frick LR, Palumbo ML, Zappia MP, Brocco MA, Cremaschi GA (2008). Inhibitory effect of fluoxetine on lymphoma growth through the modulation of antitumor T-cell response by serotonin-dependent and independent mechanisms.. Biochem Pharmacol.

[51] Gil-Ad I, Zolokov A, Lomnitski L, Taler M, Bar M (2008). Evaluation of the potential anti-cancer activity of the antidepressant sertraline in human colon cancer cell lines and in colorectal cancer-xenografted mice.. Int J Oncol.

[52] Jia L, Shang Y-Y, Li Y-Y (2008). Effect of antidepressants on body weight, ethology and tumor growth of human pancreatic carcinoma xenografts in nude mice.. World J Gastroenterol.

[53] Kelvin AS, Mitchell ID, White DJ (1989). General and genetic toxicology of paroxetine.. Acta Psychiatr Scand.

[54] Merry S, Hamilton TG, Flanigan P, Freshney RI, Kaye SB (1991). Circumvention of pleiotropic drug resistance in subcutaneous tumours in vivo with verapamil and clomipramine.. Eur J Cancer.

[55] Sauter C (1989). Cytostatic activity of commonly used tricyclic antidepressants.. Oncology.

[56] Serafeim A, Holder MJ, Grafton G, Chamba A, Drayson MT (2003). Selective serotonin reuptake inhibitors directly signal for apoptosis in biopsy-like Burkitt lymphoma cells.. Blood.

[57] Shoyab M, Todaro GJ, Tallman JF (1982). Chlorpromazine and related antipsychotic tricyclic compounds competitively inhibit the interaction between tumor-promoting phorbol esters and their specific receptors.. Cancer Lett.

[58] Slamon ND, Ward TH, Butler J, Pentreath VW (2001). Assessment of DNA damage in C6 glioma cells after antidepressant treatment using an alkaline comet assay.. Arch Toxicol.

[59] Sonier B, Arseneault M, Lavigne C, Ouellette RJ, Vaillancourt C (2006). The 5-HT2A serotoninergic receptor is expressed in the MCF-7 human breast cancer cell line and reveals a mitogenic effect of serotonin.. Biochem Biophysical Res Comm.

[60] Toth B (1975). Synthetic and naturally occurring hydrazines as possible cancer causative agents.. Cancer Res.

[61] Tsuruo T, Iida H, Nojiri M, Tsukagoshi S, Sakurai Y (1982). Potentiation of chemotherapeutic effect of vincristine in vincristine resistant tumor bearing mice by calmodulin inhibitor clomipramine.. J Pharmacobiodyn.

[62] Tucker WE (1983). Preclinical toxicology of bupropion: an overview.. J Clin Psychiatry.

[63] Tutton PJ, Barkla DH (1976). A comparison of cell proliferation in normal and neoplastic intestinal epithelia following either biogenic amine depletion or monoamine oxidase inhibition.. Virchows Arch B Cell Pathol.

[64] Tutton PJ, Barkla DH (1982). Influence of inhibitors of serotonin uptake on intestinal epithelium and colorectal carcinomas.. Br J Cancer.

[65] Volpe DA, Ellison CD, Parchment RE, Grieshaber CK, Faustino PJ (2003). Effects of amitriptyline and fluoxetine upon the in vitro proliferation of tumor cell lines.. J Exp Ther Oncol.

[66] Wright SC, Zhong J, Larrick JW (1994). Inhibition of apoptosis as a mechanism of tumor promotion.. FASEB J.

[67] Rosetti M, Frasnelli M, Tesei A, Zoli W, Conti M (2006). Cytotoxicity of different selective serotonin reuptake inhibitors (SSRIs) against cancer cells.. J Exp Ther Oncol.

[68] Arimochi H, Morita K (2008). Desipramine induces apoptotic cell death through nonmitochondrial and mitochondrial pathways in different types of human colon carcinoma cells.. Pharmacology.

[69] Xia Z, Bergstrand A, DePierre JW, Nässberger L (1999). The antidepressants imipramine, clomipramine, and citalopram induce apoptosis in human acute myeloid leukemia HL-60 cells via caspase-3 activation.. J Biochem Mol Toxicol.

[70] Chou C-T, He S, Jan C-R (2007). Paroxetine-induced apoptosis in human osteosarcoma cells: activation of p38 MAP kinase and caspase-3 pathways without involvement of [Ca2+]i elevation.. Toxicol Appl Pharmacol.

[71] Stepulak A, Rzeski W, Sifringer M, Brocke K, Gratopp A (2008). Fluoxetine inhibits the extracellular signal regulated kinase pathway and suppresses growth of cancer cells.. Cancer Biol Ther.

[72] Spanová A, Kovárů H, Lisá V, Lukásová E, Rittich B (1997). Estimation of apoptosis in C6 glioma cells treated with antidepressants.. Physiol Res.

[73] Cloonan SM, Drozgowska A, Fayne D, Williams DC (2010). The antidepressants maprotiline and fluoxetine have potent selective antiproliferative effects against Burkitt lymphoma independently of the norepinephrine and serotonin transporters.. Leuk Lymphoma.

[74] Lee CS, Kim YJ, Jang ER, Kim W, Myung SC (2009). Fluoxetine induces apoptosis in ovarian carcinoma cell line OVCAR-3 through reactive oxygen species-dependent activation of nuclear factor-jB.. Basic Clin Pharmacol Toxicol.

[75] Lin C-J, Robert F, Sukarieh R, Michnick S, Pelletier J (2010). The antidepressant sertraline inhibits translation initiation by curtailing mammalian target of rapamycin signaling.. Cancer Res.

[76] Brandes LJ (2005). Hormetic effects of hormones, antihormones, and antidepressants on cancer cell growth in culture: in vivo correlates.. Crit Rev Toxicol.

[77] Cosgrove L, Krimsky S, Vijayaraghavan M, Schneider L (2006). Financial ties between DSM-IV panel members and the pharmaceutical industry.. Psychother Psychsom.

[78] Cosgrove L, Bursztajn H, Krimsky S, Anaya M, Walker J (2009). Conflicts of interest and disclosure in the American Psychiatric Association's clinical practice guidelines.. Psychother Psychsom.

[79] Spiegel D, Giese-Davis J (2003). Depression and cancer: mechanisms and disease progression.. Biol Psych.

[80] Calabrese EJ (2005). Cancer biology and hormesis: human tumor cell lines commonly display hormetic (biphasic) dose responses.. Crit Rev Toxicol.

[81] Calabrese EJ, Baldwin LA (2001). U-shaped dose-responses in biology, toxicology, and public health.. Annu Rev Public Health.

[82] Bekelman JE, Li Y, Gross CP (2003). Scope and impact of financial conflicts of interest in biomedical research: a systematic review.. JAMA.

[83] Smith R (2005). Medical journals are an extension of the marketing arm of pharmaceutical companies.. PLoS Med.

[84] Angell M (2000). Is academic medicine for sale?. N Engl J Med.

[85] Brown WR, Gallo M, Scheuplein J, Van der Heijden KA (1991). Implication of the reexamination of the liver sections from the TCDD chronic rat bioassay.. Biological basis for risk assessment of dioxins and related compounds.

[86] Douglas H (2000). Inductive risk and values in science.. Philos Sci.

[87] Douglas H, Kincaid H, Dupré J, Wylie A (2007). Rejecting the ideal of value-free science.. Value-free science?: ideals and illusions.

[88] Fugh-Berman A (2005). The corporate coauthor.. J Gen Intern Med.

[89] Rising K, Bacchetti P, Bero L (2008). Reporting bias in drug trials submitted to the Food and Drug Administration: a review of publication and presentation.. PLoS Med.

[90] Sheldon T (2005). Focus on the funding and production of evidence rather than its publication.. PLoS Med.

[91] Thompson DF (2009). The challenge of conflict of interest in medicine.. Z Evid Fortbild Qual Gesundhwes.

[92] Falit BP (2007). Curbing industry sponsor's incentive to design post-approval trials that are suboptimal for informing prescribers but more likely than optimal designs to yield favorable results.. Seton Hall Law Rev.

[93] U.S. Preventive Services Task Force (2002). Screening for breast cancer: recommendations and rationale.. Ann Intern Med.

[94] Dunikowski LG (2005). EMBASE and MEDLINE searches.. Can Fam Physician.

[95] Kelly CM, Juurlink DN, Gomes T, Duong-Hua M, Pritchard KI (2010). Selective serotonin reuptake inhibitors and breast cancer mortality in women receiving tamoxifen: a population based cohort study.. BMJ.

