# Supplementary material for: Antidepressants and Breast and Ovarian Cancer Risk: A Review of the Literature and Researchers' Financial Associations with Industry
Source: PLoS One. 2011 Apr 6;6(4):e18210. doi: 10.1371/journal.pone.0018210 (PMC3071810; doi:10.1371/journal.pone.0018210)
Supplement: Text S1 — Search strategy. (DOC) [file pone.0018210.s001.doc]

Text S1 Search strategy

Search terms and word combinations are listed below. Every combination was entered in each database with the identified limits. With each combination the screens were used and the articles were retrieved for further review. The number of articles and/or studies was documented in Figure 1.

MEDLINE (via ISI Web of Knowledge), PSYCINFO, Science Citations Index, (no year limits, through November 2010) and the Cochrane database of Controlled Trials Register (no year limits through November 2010).

Search strategy

1. carcinogenic and antidepressant
2. neoplastic and mianserin
3. mianserin and adverse effects
4. desipramine, clomipramine, lofepramine and apoptotic, mitogenic, neoplastic
5. breast cancer and fluoxetine, sertraline
6. nortriptyline, protriptyline, imipramine, and ovarian cancer
7. imipramine and mutagenic, clastogenic
8. antidepressants, SSRIs, tricyclics, MAO inhibitors and cancer
9. antidepressants, SSRIs, tricyclics, MAO inhibitors and carcinogenic
10. fluoxamine and carcinogenic
11. venlafaxine and neoplastic
12. venlafaxine, amitriptyline, nortriptyline, protriptyline, imipramine, and genotoxicity
13. ovarian cancer and antidepressant
14. breast and ovarian cancer and antidepressant
15. carcinoma and fluoxetine
16. Citalopram and Escitalopram and Fluoxetine and cancer and carcinoma
17. Fluvoxamine and cancer and carcinoma and Paroxetine
18. Ovarian Cancer and Antidepressants
19. Carcinogenic and Antidepressants
20. metastatic ovarian carcinoma and antidepressants
21. Fluoxetine and Women and antidepressants
22. SSRI and Cancer
23. Fluoxetine and Cancer
24. Tricyclics and Cancer
25. Antidepressants and Cancer
26. Sertraline and carcinogenic
27. MAOI and carcinogenic
28. nortriptyline, protriptyline, imipramine and carcinoma
29. Isocarboxazid and Moclobemide, Phenelzine, and carcinogenic
30. Tranylcypromine, Selegiline, Iproniazid and mutagenic, clastogenic
31. Pargyline, Trazodone, Clomipramine and genotoxicity
