# Supplementary material for: Antidepressants and Breast and Ovarian Cancer Risk: A Review of the Literature and Researchers' Financial Associations with Industry
Source: PLoS One. 2011 Apr 6;6(4):e18210. doi: 10.1371/journal.pone.0018210 (PMC3071810; doi:10.1371/journal.pone.0018210)
Supplement: Table S1 — Summary of epidemiological studies on antidepressants and cancer risk. (DOC) [file pone.0018210.s002.doc]

Table S1 Summary of epidemiological studies on antidepressants and cancer risk

| **Reference** | **Time and place** | **Study design** | **Participants** | **Drug type and information** | **Exposure assessment** | **Exposure definition** | **Results** | **Conclusion** |
| --- | --- | --- | --- | --- | --- | --- | --- | --- |
| Chien (25) | 1997-1999, US | Population-based case-control study | 975 breast cancer cases and 1007 controls  Age 65-79 | TCA, SSRI, Triazolopyridines (nefazodone and trazodone). Length of use. No dosage information. | Self report | Ever use of an AD drug in the category for at least 3 months | Ever use of AD and breast cancer: OR=1.2 (0.9-1.6). TCA: OR=1.2 (0.8-1.8). SSRI: OR= 1.2 (0.8-1.8). Triazolopyridines: OR=1.7 (0.9-3.3). Ever use of SSRI and ER+/PR- breast cancer: OR = 1.8 (1.1-3.6)  ER-PR- breast cancer: OR = 2.0 (1.1-3.8) | Yes. Moderately significant for SSRI on ER-PR-, ER+/PR- breast cancers. |
| Coogan (26) | 1976-1988, US | Hospital based case-control study | 748 epithelial ovarian cancer cases, 1496 cancer controls and 1496 non-cancer controls Median age 50-59 | SSRI, TCA, Phenothiazines, Benzodiazepines. No information on dosage or duration of use | Self report | Regular use of AD: use of AD at least 4 days a week for a duration of at least 4 weeks | SSRI: cases vs cancer controls: OR=0.8 (0.3-2.3), vs non-cancer controls OR=3.0 (0.8-10.5). TCA: cases vs cancer controls: OR=0.8 (0.4-1.5), vs non-cancer controls OR=0.7 (0.4-1.3) | No |
| Coogan (27) | 1988-2002, US | Hospital based case-control study | 2138 invasive breast cancer cases, and 2858 controls Age 24-73 | Overall use of SSRI and specific SSRI: fluoxetine, sertraline, and paroxetine. Duration of use (<1 year, 1-2, 2-4, ≥4 years)  No dosage information | Self report | Regular use of SSRI: use of SSRI at least 4 days a week for at least 3 continuous months at least 1 year before admission | SSRI: OR=1.1 (0.8-1.7). ≥ 4 years: OR=0.7 (0.4-1.5). Continuing use: OR=1.2 (0.8, 1.8) | No |
| Coogan (28) | 1990-2006, US | Hospital based case-control study | 820 invasive breast cancer cases, and 2852 controls  Age 25-79 | Regular use of SSRI. No information on duration and dosage of use | Self report | Regular use of SSRI: use of SSRI at least 4 days a week for at least 3 continuous months at least 1 year before admission | OR=0.89 (0.62-1.29). Among aged 55+: OR=1.43 (0.76-2.69) | No |
| Cotterchio (1) | 1995-1996, Canada | Population-based case-control study | 701 breast cancer cases, 702 controls  Age 25-74 | TCA, SSRI, MAOI, atypical  Specific AD. Duration of use. No dosage information | Self report | Use of AD for at least 2 weeks | Any AD use: OR=1.2 (0.8, 1.7). Use of TCA for ≥25 months: age-adjusted OR=2.5, 1.2-5.1 multivariate OR=2.1; 0.9-5.0.. Use of SSRI for ≥25 months: age-adjusted OR=0.9, 0.3-2.6; multivariate OR=0.7; 0.2-2.2. Paroxetine: age-adjusted OR=9.1, 1.2-72.5; multivariate OR=7.2 (0.9, 58.3) | Yes. Significant. for use of TCA ≥25 months and for paroxetine |
| Dalton (29) | 1989-1995, Denmark | Population based retrospective cohort study | 966 patients with any cancer among 30, 807 AD users. Median age = 57 | TCA only, TeCA only, SSRI only, MAOI only, Mixed use. Frequency of use  Number of prescriptions (1, 2-4, 5 and more) | Electronic record (Prescription database) | Filled prescription at least 1 year prior to the index date | Any AD: SIR=1.0(1.0-1.1). TCA: SIR=1.0 (0.9-1.1). TeCA: SIR=1.3 (1.1-1.7). SSRI: SIR=0.8 (0.7-1.0). MAOI: SIR=0.6 (0.1-1.6). Mixed: SIR=1.1 (0.9-1.2). When ≥5 prescriptions: TCA and non-Hodgkins lymphoma: SIR=2.5 (1.4-4.2). TeCA and total cancer : SIR=1.6 (1.2-2.1) | Yes. Significant for regular use of TCA on non-Hodgkins lymphoma and TeCA on total cancer |
| Danielson (16) | 1977-1980, US | Retrospective cohort study | 302 breast cancer cases in 184,438 person years age 35-74 | TCA, diazepam, digitalis glycosides, medroxyprogesterone, methyldopa, reserpine, phenothiazines, spironolactone, metronidazole, etc. No information on duration or dosage | Electronic record (pharmacy database ) | AD dispense within 6 months before the diagnosis of breast cancer | TCA: RR=0.5 (90% CI 0.3-0.8) Reserpine: RR=1.7 (0.9-3.3) | No |
| Davis (42) | 1992-1995, US | Population-based case control study | 622 breast cancer cases and 647 controls. Age 20-74 | Overall use of AD. Duration of use (<5 & 5-10 years). Recency of use (>2 and ≤2 years of diagnosis). No dosage info. | Self report | Regular use of AD: taking AD at least four days a week for at least 6 months during 10 years prior to the index date | Regular use: OR=1.3 (0.8-1.9). 5-10 years of use: OR =1.4 (0.7-2.7). Within 2 years of diagnosis: OR=1.3 (0.8-2.1) . Post menopausal: OR = 1.8 (1.0-3.0) | Yes. Significant for post menopausal women |
| Dublin (18) | 1981-1997, US | Population based case-control study | 314 epithelial ovarian cancer cases and 790 controls. Age: 35-79 | Overall use of AD  Benzodiazepine. Number or prescription  Number of pills dispensed)  Continuity of use (never, not continuous for over 6 months, continuous for 6 months) | Electronic record (pharmacy database) | Filled prescription of AD in 1.5 years prior to the reference date | Overall use of AD: 2 prescription within 6 months: OR=0.71 (0.47-1.10). Continuous use for over 6 months OR = 0.64 (0.36-1.10)  Benzodiazepine: 2 prescription within 6 months: OR=0.70 (0.47-1.00). Continuous use for over 6 months OR = 0.53 (0.15-1.90) | No |
| Fulton-Kehoe (43) | 1990-2001, US | Population based case-control study | 2904 breast cancer cases and 14396 controls. Age 30-79, median age 60-69 | Overall use of AD  TCA, SSRI, atypical. Number of prescription refills. No dosage info | Electronic record (pharmacy database) | Filled prescription of AD at least twice in a 6-month interval at least 1 year prior to the index date | Any AD: OR=1.04 (0.94-1.16). TCA: OR=1.06 (0.94-1.19). SSRI: OR=0.98 (0.8-1.18). Atypical: OR=0.95 (0.78-1.16) | No |
| Gonzalez- Perez (30) | 1995-2001, UK | Nested case-control study | 3708 breast cancer cases, and 20000 controls. Age 30-79 | SSRI, TCA, Other AD. Duration of use (<1 year, 1-3 years, and >3 years)  Dosage (medium to low, and high dose) | Electronic record (General Practice Research Database) | Filled prescription of AD before the index date | SSRI:OR= 0.98 (0.81-1.19). TCA: OR=0.86 (0.73-1.00). Other AD: OR=1.15 (0.82 – 1.61). > 3 years: SSRI: OR=0.56 (0.27-1.18). TCA: OR=0.83 (0.62-1.09). Other AD: OR=1.26 (0.67-2.37). High dose: SSRI: OR=0.95 (0.46-1.96). TCA: OR=1.11 (0.71-1.71) | No |
| Haque (31) | 1995-2000, US | Retrospective cohort study | 635 breast cancer cases among 109004 AD users  Age 18-106, mean age between 43-53 | SSRIs, Paroxetine, fluoxetine, Atypical AD. Duration of use (<2 and ≥2 years)  No dosage info | Electronic record (pharmacy database) | Filled prescription of AD at least 1 year prior to the diagnosis | Paroxetine only: RR=1.16 (0.98-1.38). SSRI only: RR= 1.14 (0.87-1.49). Paroxetine or SSRI: RR=1.01 (0.67-1.51) | No |
| Harlow (4) | 1978-1981 and 1984-1987, US | Population based case-control study | 450 epithelial ovarian cancer (cases) and 454 controls. Age 18-80 | Overall use of AD (including Elavil, Imipramine, Limbritol, Tofranil, Triavil, etc). No information on dosage or duration | Self report | Prior use of AD exceeding1-6 months | Use of AD and ovarian cancer: OR=2.1 (0.9-4.8; Among age < 50 at first use: OR=3.5 (1.3-9.2) | Yes. In <50 years of age at first use |
| Harlow (32) | 1992-1997, US | Population based case-control study | 563 epithelial ovarian cancer (cases) and 523 controls.  Average 49-50 | Use of AD (TCA, MAOI, and SSRI) and other psychotropic medications. Timing and length of medication. No dosage info. | Self report | Continuous use of psychotropic drug for 6 months or longer, during at least 1 year prior to the index date | Any use of psychotropic medication: OR=1.6; 1.1-2.3. SSRI: OR=1.0 0.4-2.1. Inhibitors of γ-amino butyric acid: OR=1.5 (0.9-2.5). Dopamine /norepinephrine reuptake inhibitors: OR=2.9 (1.3-6.4) | Yes |
| Haukka (41) | 1998-2005, Finland | Population based prospective cohort study | 19365 any type cancer cases among 837176 people  Average age: 46-48 | SSRI, Non-SSRI. Duration of use. No dosage information | Electronic record  (nationwide prescription database) | Filled prescription at least once between 1998 and 2005, and no AD use in pervious 3 years | Over 4 years of SSRI and all cancers: HR=1.08 (0.93-1.26) . Over 4 years use of SSRI and breast cancer: HR=1.53 (1.14-2.05), but additional analyses showed no association | No |
| Kato (33) | 1985-1991, US | Prospective cohort study | 566 breast cancer, 47 ovarian and 67 endometrial cancers among 15270 women | Overall use of AD. No information on dosage or duration | Self report | Use of AD in 4 weeks preceding enrollment in the study | AD and breast cancer: RR= 1.75 (1.06-2.88). AD and breast/ ovarian/endometrial cancers: RR=1.80 (1.15-2.81) | Yes |
| Kelly (2) | 1977-1996, US | Hospital-based case-control study | 5814 breast cancer cases, 5095 cancer controls and 5814 noncancer controls. Age 18-69, median age 51 | TCA, SSRI, other AD. Duration of use (<1, 1-4. 5-9, ≥10 years). Recency of use (<1, 1-4. 5-9, ≥10 years since last use). No dosage info | Self report | Use at least 4 days a week for at least 4 weeks beginning more than 1 year before admission | TCA: cases vs cancer controls RR=1.1 (0.8-1.5) vs non cancer controls: RR=0.8 (0.6-1.0). SSRI: cases vs cancer controls RR=1.6 (0.8-3.2) vs non cancer controls: RR=1.5 (0.8-2.8)  1-2 years of use: RR=2.0 (1.0-4.3). Recent use (< 1 year since last use): RR=1.8 (1.0, 3.3) | Yes. Significant for recent use of SSRI |
| Moorman (34) | 1999-2003, US | Population-based case-control study | 593 epithelial ovarian cancer cases, and 628 controls. Age 20-74 | Any use of AD  SSRI only, TCA only, other types of AD, multiple types of AD. Duration of use (≤1 year, 2-3, 4-5, 6-10, >10 years). No dosage info | Self report | Use of AD for more than 6 months | Any use of AD:  OR=0.9 (0.7-1.2)  >10 years of use: OR=0.7 (0.4-1.4)  SSRI only: OR= 1.0 (0.7-1.5)  >5 years: OR=1.2 (0.5-2.2)  TCA only: OR= 0.5 (0.2-1.1)  Other AD: OR=0.8 (0.4-1.5)  Multiple types: OR=0.9 (0.5-1.6) | No |
| Moorman (35) | 1996-2000, US | Population-based case-control study | 938 invasive breast cancer cases, 771 controls  And 507 carcinoma in situ cases and 455 controls. Age 20-74 | TCA, SSRI, atypical (e.g. trazodone, bupropion), MAOI, lithium or multiple types. No information on dosage or duration | Self report | 3 or more months of use in past 10 years | Any use of TCA: OR= 1.0; 0.7-1.2. Use of TCA for 36-59 months OR=1.6; 0.8-2.9. Use of TCA: OR=1.0; 0.7-1.5, use of TCA for ≥36 months: OR=0.7; 0.3-1.7. Use of SSRI: OR=1.0; 0.7-1.5. Use of SSRI for ≥ 36 months OR= 2.2; 0.8-6.3 | No |
| Sharpe (36) | 1981-1995, Canada | Population-based case-control study | 5882 breast cancer cases, 23517 controls. Age ≥35 | Overall use of TCA and two categories of TCA: genotoxic and non genotoxic. No information on duration or dosage | Electronic record  (prescription drug plan database) | Filled prescription for TCA before the index date | Heavy exposure to overall TCA 11-15 years later: RR=2.02 (1.34-3.04). Heavy exposure to genotoxic TCA 11-15 years later: RR=2.47 (1.37-4.40). Longest duration (71-100%) of genotoxic TCA use 11-15 years later: 2.39 (1.30-4.39) | Yes. Significant in heavy exposure and long duration of use of genotoxic TCA |
| Steingart (24) | 1996-1998, Canada | Population based case-control study | 3133 breast cancer cases, and 3062 controls. Age 25-74 | SSRI, TCA, MAOI, atypical. Duration of AD use. Recency of last AD use  Time since first AD use. No dosage info | Self report | Taken daily for at least 2 months at least 1 year prior to the index date | Overall use of AD: OR=1.20 (0.96-1.51). SSRI: OR=1.32 (0.97-1.80). TCA: OR=1.10 (0.83-1.45). MAOI: OR= 0.80 (0.27-2.4). Atypical: OR=1.04 (0.5-2.16) | No |
| Tamim (37) | 1981-2000, Canada | Population based case-control study | 7330 breast cancer cases, and 29320 controls. Average age 61, range 20-83 | Two categories of TCA: genotoxic and non genotoxic  No information on dosage or duration of drug use | Electronic record  (outpatient prescription drug database) | Filled prescription for TCA at least 1 year prior to the index date | Use of genotoxic TCA 2-5 years prior to the index date: RR=1.34, 1.07-1.68 for medium dose and RR=1.32, 1.04-1.67 for high dose. | Yes |
| Wallace (38) | 1974-1978, US | Hospital based case-control study | 151 breast cancer cases and 151 hospital controls. Average age 58 | Overall use of AD  Amitriptyline, nortriptyline, desipramine, and phenelzine  No information on dosage or duration | Self report | Use of AD regularly for longer than 1 month | Use of AD: adjusted RR=2.84 (p<0.04), but it lost significance after adding interaction term of AD use and socioeconomic status: RR=1.62, P>0.2 | No |
| Wang (17) | 1989-1991, US | Retrospective cohort study | 319 breast cancer cases among 38273 AD users and 252 cases among 32949 non-AD users. Age ≥20 | Overall use of AD  Specific TCA (Amitriptyline, Fluoxetine, Doxepin, Trazodone, etc)  Duration of use (quartile)  Dose (quartile) | Electronic record (prescription database) | Filled prescription for ADs from the index date to the end point (breast cancer diagnosis, July 1^st^ 1991, or death) | AD: HR=1.04 (0.87-1.25). TCA: HR= 1.09 (0.92-1.31). High dose of AD: HR=1.02 (0.77-1.35). Long duration of AD use: HR=1.00 (0.75-1.33) | No |
| Weiss (39) | 1988-1994, US | Nested  case-control study | 1467 patients with breast cancer, colon cancer or malignant melanoma. Each patient was matched with 5 controls. Average age 62 years | Overall use of antihistamines or ADs. Number of prescription filled. No dosage information | Electronic record  (Pharmacy dispensing database) | Filled prescription for ADs or antihistamines from the date of diagnosis to the end of the follow-up period | Use of antihistamines or Ads was not associated with tumor recurrence (OR=0.97; 0.52-1.78) or development of second primary cancer (OR=0.94; 0.51-1.77). Number of prescriptions filled was not associated with tumor recurrence (1-2 refills: 0.88; 0.39-2.04; 3-7 refills: 1.40; 0.57-4.13; 8 or more refills: 1.54; 0.41 – 5.78) or development of second primary tumors (1-2 refills: 1.04; 0.50 -2.17; 3-7 refills: 0.83; 0.22-3.06; 8 or more refills: 0.71; 0.16-3.26) | No |
| Wernli (40) | 2003-2006, US | Population-based case control study | 2908 breast cancer cases and 2927 controls. Age 20-69 | SSRI, TCA, NDRI, SNRI. Ever, former, current and never use. Duration of use: never, <2, 2-4, 5-9 and ≥ 10 years | Self report | use of AD for 3 months or more | Ever use of AD: OR=0.89 (0.78-1.01). Former use: OR=0.82 (0.66-1.01). Current use: OR=0.92 (0.80-1.07. Duration ≥ 10 years: OR=0.87 (0.66-1.15). Ever use of SSRI: OR=0.85 (0.72-1.00). Ever use of TCA: OR=0.90 (0.60-1.35). Ever use of NDRI: OR=0.92 (0.62-1.38). Ever use of SNRI: OR=1.18 (0.72-1.92) | No |

CI= confidence interval ; AD=antidepressant; TCA= tricyclic antidepressant; TeCA=tetracyclic antidepressant; SSRI= selective serotonin reuptake inhibitor; MAOI = monoamine oxidase inhibitor; NDRI =Norepinephrine-dopamine reuptake inhibitor; SNRI= serotonin-norepinephrine reuptake inhibitor; HR= hazard ratio; OR = odds ratio; RR= relative risk; SIR=standardized incidence ratio; PR = progesterone receptor; ER = estrogen receptor
