# Supplementary material for: Antidepressants and Breast and Ovarian Cancer Risk: A Review of the Literature and Researchers' Financial Associations with Industry
Source: PLoS One. 2011 Apr 6;6(4):e18210. doi: 10.1371/journal.pone.0018210 (PMC3071810; doi:10.1371/journal.pone.0018210)
Supplement: Table S2 — Results and study design of epidemiological studies. (DOC) [file pone.0018210.s003.doc]

Table S2 Results and study design of epidemiological studies

| **Study Design** | **Positive Association** | | **Total** |
| --- | --- | --- | --- |
|  | **Yes** | **No^*^** |  |
| Case control study | 8 | 12 | 20 |
| Cohort study | 2 | 4 | 6 |
| Total | 10 | 16 | 26 |

* In 6 studies authors noted and expressed concerns over elevated risks, although the increased risk did not reach statistical significance.
