# Supplementary material for: Antidepressants and Breast and Ovarian Cancer Risk: A Review of the Literature and Researchers' Financial Associations with Industry
Source: PLoS One. 2011 Apr 6;6(4):e18210. doi: 10.1371/journal.pone.0018210 (PMC3071810; doi:10.1371/journal.pone.0018210)
Supplement: Table S3 — Summary of pre-clinical studies on antidepressants and cancer risk. (DOC) [file pone.0018210.s004.doc]

Table S3 Summary of pre-clinical studies on antidepressants and cancer risk

| **Reference** | **Time and place** | **Study design** | **Drug type and information** | **Exposure definition** | **Results** | **Conclusion** |
| --- | --- | --- | --- | --- | --- | --- |
| Abdul (45) | 1995, US | Assess effect of serotonin (ST) uptake inhibitors (SUIs) on growth of 3 human prostatic carcinoma cell lines | 6-nitroquip-azine, zimeli-dine and fluoxetine. *In vivo* growth: 40 ìg/day x 6 wks | n/a | All 3 SUIs inhibited proliferation of the 3 human prostatic carcinoma cell lines (PC-3, DU-145, and LNCaP) in a dose-dependent manner (fluoxetine > zimelidine > 6-nitroquipazine); also, all 3 SUIs blocked ST uptake by the prostatic carcinoma cell lines. | No*. Significant for dose-dependent inhibition of proliferation of 3 human prostatic carcinoma cell lines |
| Arimochi (68) | 2008, US | Assess effect of desipramine on growth of human colon carcinoma cell lines HT29 and HCT116 | Desipramine (DMI). *In vivo* growth: 50-75 ìmole/l x different time periods | n/a | The present study shows that desipramine (DMI) causes the  nonoxidative cytotoxic effect on human colon carcinoma HT29 cells through the nonmitochondrial apoptotic pathway, and also that it causes the nonoxidative apoptotic damage to colon carcinoma HT116 cells probably through the novel mitochondrial pathway. | No*. Significant for dose-dependent DMI-induced cytotoxic (nonoxidative apoptotic damage) to 2 human colon carcinoma cell lines (HT29 and HCT116) |
| Basso (46) | 1992, Argentina | Assess for possible anti-tumoral effects of imipramine (IMI) in rats who had been inoculated with DMBA-induced sarcoma cells and who were exposed to a chronic variable stress procedure (CVS) and/or to the immunosuppressant drug cyclosporine (CS) | Imipramine (IMI). Dose: 10 mg/kg i.p. Duration: 10 mg/kg x 14 days | Tumoral cells were derived from a sarcoma originally induced by 9,10-dimethyl-1,2-benzanthracene (DMBA). Tumoral cells were obtained from a singeneic Wistar donor rat bearing this kind of tumor induced by carcinogen. To induce tumor formation, 5 x 1~ cells contained in 0.1 ml of medium were implanted s.c. in both lateral sides of the abdominal area. | Chronically stressed rats which were concurrently injected with the antidepressant drug IMI showed a significant reduction in tumoral incidence relative to animals exposed to CVS without antidepressant treatment. However, IMI did not modify tumoral growth that was precipitated by the immunosuppressive agent cyclosporine (CS). | No*. The psychological experience of “depression” somehow lowers the capacity to defend against tumor growth, while the modification of this experience by the antidepressant agent imipramine (IMI) normalizes the ability to resist this neoplastic process. |
| Bendele (47) | 1992, US | In a test for possible carcinogenicity, fluoxetine HCl was administered to rats and mice *ad libitum* via continuously administered mash at dietary doses for 24 months. | Fluoxetine HCl. Dose: concentrations of 0.0, 0.001, 0.0045, or 0.02% for rats and 0.0, 0.001, 0.004, or 0.01% for mice in the control and respective treatment groups. Duration: 24 months | n/a | There was no evidence of an increased incidence of any type of unusual or commonly occurring spontaneous neoplasm in either rats or mice. There were statistically significant decreases in a few commonly occurring neoplasms. | No. The data reported provide evidence that fluoxetine is neither a complete carcinogen nor a tumor promoter**.** Also, there were statistically significant decreases in a few commonly occurring neoplasms. |
| Brambilla (48) | 1982, Italy | 4 *in vivo* or *in vitro* studies were carried out to determine whether 5 hydrazine-derivative antidepressants displayed tumorigenic activity. | Phenelzine, nialamide, mebanazine, isocarboxazid, and iproniazid. Dose: *in vivo*: single administration of 2 x LD50, reduced to LD50 as necessary; daily administration for 5 (sometimes 15) days of 1/3 LD50 | Relative potencies were calculated as the ratio of the investigated effect to the corresponding molar dose of compound within each experimental model. | All drugs except iproniazid produced DNA fragmentation; all drugs except mebanazine were weak inducers of sister chromatid exchanges (SCEs); phenelzine and nialamide elicited base-pair substitutions and mebanazine elicited frameshift errors; and phenelzine, nialamide and mebanazine were positive in a DNA repair bacterial test. | Yes. Isocarboxazid displayed the highest activity in damaging DNA and increasing the frequency of SCEs; nialamide was the most potent bacterial mutagen (Ames Test); and phenelzine exhibited the greatest toxicity to repair-deficient *E. Coli* strains. All hydrazine derivatives tested thus far have been found to be tumorigenic in rodents – these results add further information on the genotoxic properties of hydrazine antidepressants. |
| Brandes (5) | 1992, Canada | *In vivo* and *in vitro* studies were carried out to determine whether amitriptyline and fluoxetine stimulate tumor growth and/or development in rodents at concentrations relevant to the treatment of human depression. | Amitriptyline and fluoxetine. Dose: equivalent human dose range, ~ 100-150 mg/day for amitriptyline and ~20-80 mg/day for fluoxetine | Mice were inoculated s.c. with C-3 fibrosarcoma cells or given i.v. or s.c. injections of BloflO melanoma cells; mammary tumors development was assessed in rats fed DMBA. | 8 of 20 amitriptyline- or fluoxetine-treated mice developed fibrosarcoma tumors by day 5, as compared to none of 20 saline controls (P < 0.002). Similarly, 20 of 21 DMBA-treated rats receiving the antidepressant drugs developed 33 mammary tumors by week 15 as compared to 5 tumors in 4 of 7 DMBA-treated rats receiving saline (/P < 0.001). For both models, tumor latency decreased 30-40% and, in the DMBA model, tumor frequency increased >2-fold in the antidepressant treated rats as compared to controls. Stimulation of fibrosarcoma growth *in vivo* correlated with a corresponding bell-shaped drug-induced increase  in DNA synthesis *in vitro*. | Yes. The demonstration that amitriptyline and fluoxetine inhibit normal lymphocyte proliferation *in vitro* and promote tumor growth in 3 of 3 rodent models *in vivo* raises the question of whether they could have a similar effect in humans. The stimulation of DNA synthesis in C-3 fibrosarcoma cells by amitriptyline and fluoxetine occurs at somewhat lower in vitro concentrations than does their inhibition of lymphocyte mitogenesis. |
| Brandes (76) | 2005, Canada | Following publication of Brandes (5), Bendele et al. (Eli Lilly laboratories) reported that in pre-clinical testing, no evidence of an in-crease in spontaneous tumors was observed in mice or rats chronically dosed for 2 years with fluoxetine. Breast and pituitary tumors were actually decreased in fluoxetine-treated rodents. However, in analyzing tumor incidence for significance, the authors employed the two-tailed Cochran–Armitage trend test for linearity. | Fluoxetine HCl. Dose: concentrations of 0.0, 0.001, 0.0045, or 0.02% for rats and 0.0, 0.001, 0.004, or 0.01% for mice in the control and respective treatment groups. Duration of administration: 24 months | n/a | Applying the Poisson exact test to analyze the same data, the author  found a significant (*p < .*02) increase in spontaneous lung tumors (benign and malignant) in mice administered low or average human equivalent-doses (10–40 mg/day) of fluoxetine, whereas a significant (*p < .*006) decrease in lung tumors occurred in mice administered high human-equivalent doses (80+ mg/day) of the drug. Significant hormetic responses at low to mid doses were also observed for adrenal tumors and lymphoreticular neoplasms. | Yes. A reconsideration of the results of the Bendele (48) study suggested a significant increase in spontaneous lung tumors (benign and malignant) in mice administered low or average human equivalent-doses (10–40 mg/day) of fluoxetine. Also, a significant decrease in lung tumors occurred in mice administered high human-equivalent doses (80+ mg/day) of the drug. Significant hormetic responses at low to mid doses were also observed for adrenal tumors and lymphoreticular neoplasms. |
| Chou (70) | 2007, Taiwan | The aim of the present study was to investigate the molecular mechanisms underlying the apoptotic pathway induced by paroxetine in osteosarcoma cells by using MG63 cells. The authors explored the cytotoxic effect of paroxetine on MG63 cells, and investigated whether apoptotic markers (such as caspase-3 activation and increase in subdiploidy nuclei), MAPKs and Ca^2+^ signaling were interacting in this event. | Paroxetine | To examine the cytotoxicity of paroxetine in human osteosarcoma cells, MG63 cells were cultured in the presence of various concentrations of paroxetine and cell viability assays were performed. | The major novel finding of the present study is that paroxetine caused apoptosis of human MG63 osteosarcoma cells in a Ca^2+^-independent manner. Paroxetine-induced cell death was accompanied by DNA fragmentation. Immunoblotting assays also detected that paroxetine could induce the activation of caspase-3. The cell viability results suggest that at concentrations greater than 50 μM, paroxetine induced concentration-dependent cell death. There were 97% cells dead in the presence of 100 μM paroxetine. The relationship between the incubation time of paroxetine and the percentage of dead cells was of high correlation. Incubation with paroxetine for 12 h could kill 37% of cells. | No*. In human MG63 osteosarcoma cells, paroxetine, at dose concentrations that could be encountered in overdosed patients, induced cell death via a Ca Ca^2+^-independent, p38 MAPK-dependent, apoptotic pathway. Although paroxetine also activated ERK and JNK MAPKs, those expressions were not involved in this apoptotic pathway. |
| Clayson (49) | 1965, Italy | This report is concerned with the carcinogenic action of 5 derivatives of hydrazine to the lungs of BALB/c/Cb/Se mice. | Hydrazine derivatives:  Phenylhydra-zine, benzoyl- hydrazide, 2-methoxyben-zoylhydrazide, 4-methoxy-benzoyl- hydrazide, and iproniazid | Drugs were administered 7 x per week in aqueous solution via stomach tube to BALB/c/Cb/Se mice. | Benzoyl hydrazide, 2-methoxybenzoyl hydrazide, and 4-methoxybenzoyl hydrazide exhibited carcinogenicity in mice by increasing the proportion of mice with tumors and the number of tumors per affected mouse.  Phenylhydrazine induced pulmonary tumors of a greater degree of malignancy. The carcinogenicity of iproniazid was judged to be equivocal. | Yes. Isoniazid is carcinogenic by virtue of its hydrazine moiety. |
| Cloonan (73) | 2010, Ireland | This study examined (a) the role of the norepinephrine transporter in the antidepressant-mediated cell death of Burkitt lymphoma across a range of antidepressants and (b) whether or not compounds that target serotonin transporters induce apoptosis independent of the serotonin transporters in Burkitt lymphoma and related malignancies. | Fluoxetine,  Sertraline,  Citalopram,  Paroxetine,  Maprotiline,  Nisoxetine,  Reboxetine,  Desipramine,  Imipramine,  Amitriptylin. Dose: 50 μM  Duration: 24 hrs. Except DG-75 cell line:  Dose: 50 μM  Duration: 72 hrs | Cells were seeded at a density of 76105 cells/5 mL and incubated with 50 mM antidepressant for 24 h except for the DG-75 cell line which was incubated for 72 h. | Apoptosis induction varied by type of antidepressant. Fluoxetine, maprotiline, and sertraline induced cell death across the range of cell lines, including the Burkitt Lymphoma cells. Other drugs had limited effect, either less potent or in selective cell lines only. Fluoxetine and maprotiline were further studied and found to have anti-proliferative potential with other malignancies and to manifest selective toxicity to malignant cells over ‘normal’ cells. Serotonin transporters and norepinephrine transporters were not found to be involved in the apoptosis induced by fluoxetine and maprotiline. Desipramine was the only norepinephrine transporter inhibitor that showed effects similar to maprotiline. | No.* Fluoxetine and maprotiline induce cell death in Burkett Lymphoma without the involvement of serotonin transporters or norepinephrine transporters. In addition, it was found that both fluoxetine and maprotiline displayed antiproliferative activities when tested with 3 breast-derived malignancies, with approximate EC50 potencies of between 5 and 30 mM in all of the cell lines. |
| Frick (50) | 2008, Argentina | The authors evaluated the influence of fluoxetine  in the *in vivo* biological behavior of a T-cell lymphoma growing on syngeneic wild type mice and on athymic (nude) mice. In addition, they analyzed the *in vitro* proliferation of tumor cells in the presence of this drug. They also studied the in vivo effects of fluoxetine on the T-cell-mediated immunity, and the expression of the antitumoral cytokines. Finally, they analyzed the *in vitro* effects of fluoxetine and serotonin upon mitogen-induced T-cell proliferation in order to better understand the mechanisms of action of fluoxetine. | Fluoxetine. Dose: 15 mg/kg/day in drinking water. Duration: 8 weeks | Intraperitoneal transplantation of LBC cells into mice resulted in an aggressive T-lymphoblastic lymphoma. The effect of fluoxetine or 5-hydroxytryptamine hydrochloride (i.e., serotonin) on LBC cells proliferation was also evaluated. | Mice that were always treated with fluoxetine displayed a markedly reduced tumor growth compared to control animals. On the other hand, when fluoxetine treatment was interrupted, tumor growth was increased, but never to values achieved by untreated animals. Finally, when treatment began after injection, tumor proliferation was partially inhibited. Additionally, mice treated with fluoxetine prior to neoplastic cells injection had a delayed latency of appearance of solid tumors. Moreover, animals treated continuously with fluoxetine had a greater survival than control animals, whereas early and late treatments resulted in intermediate values. Although the most remarkable difference was found when animals were continuously (Always) treated with fluoxetine, it is worth noting that antidepressant treatment after tumor development significantly increased survival rate. These mice also displayed an increase of the antitumor cytokines. Given that fluoxetine does not act directly on lymphoma cells, this antidepressant could be acting at the immune system level to inhibit tumor growth. | No*. Fluoxetine inhibits tumor growth, delays its appearance, and extends survival of mice. All fluoxetine treatments ameliorate tumor progression in terms of survival. Survival was improved in mice treated with fluoxetine after tumor injection. Fluoxetine-induced modulation of T-cell mediated immunity is a crucial component of the mechanism that leads to an inhibition of tumor growth. Finally, the results show that fluoxetine is effective in delaying the tumor progression by regulating the antitumor immune response by serotonin-dependent and independent mechanisms, pointing to a potential therapeutic action of fluoxetine on cancer treatment. |
| Gil-Ad (51) | 2008, Israel | The authors tested the effect of different antidepressants on cell viability and proliferation of human colorectal carcinoma cell lines HT29 and the multi-drug resistant (MDR) LS1034. *In vivo*, they used CD1 nude mice xenografted subcutaneously with HT29 cells to evaluate whether sertraline and paroxetine significantly inhibited tumor growth. | Sertraline, paroxetine, fluoxetine, clomipramine and reboxetine. *In vitro* dose: 5-30 μM x 24 h). *In vivo* dose: 15 mg/kg i.p. each) Duration: Therapy began on day 8 after cell inoculation (sertraline, paroxetine or 5-FU twice a week on the first three weeks, followed by three times a week from the 3rd week. | *In vivo*, the authors used CD1 nude mice xenografted subcutaneously with HT29 cells. On day 8, after cell inoculation sertraline or paroxetine (15 mg/kgx3/ week i.p.) were administered to animals (6/group), which were monitored weekly (for 5 weeks) for tumor volume and body weight. | The authors tested the effect of different antidepressants on cell viability and proliferation of human colorectal carcinoma cell lines HT29 and the multi-drug resistant (MDR) LS1034. The SSRIs, sertraline and paroxetine, induced a dose-dependent inhibition of cell viability and proliferation in the two cell lines (IC50 8-15 μM). Sertraline showed the most potent inhibition of viability of HT29 cells with IC50 of 14.7 μM followed by paroxetine, clomipramine and fluoxetine, while the noradrenaline reuptake inhibitor, reboxetine, showed no effect on cell survival or apoptosis. When compared to cytotoxic agents e.g. doxorubicin, vincristine and 5-fluorouracil, the SSRIs showed comparable activity (HT29) or a superior effect (LS1034). *In vivo*, they used CD1 nude mice xenografted subcutaneously with HT29 cells. Sertraline, though not paroxetine, significantly inhibited tumor growth. | No*. The results suggest a proapoptotic activity for the active SSRIs accompanied by mitogen-activated protein kinase cascade activation and Bcl-2 inhibition. Collectively, the results suggest that the widely-used antidepressant, sertraline, possesses a potential anti-tumor activity, which circumvents the MDR mechanism. Since SSRI therapy is frequently indicated in cancer patients, the use of sertraline in colon cancer patients with co-morbidity of depression seems attractive. |
| Hilakivi-Clarke (13) | 1993, US | In rats, neonatal clomipramine administration induces subsequent behavioral abnormalities that closely resemble those seen in human endogenous depression. Early postnatal handling, on the other hand, improves subsequent ability to cope with stress in rodents. The present study examined whether early clomipramine treatment or handling influences the growth of 7,12-dimethylbenz(a)an-thracene (DMBA)-induced mammary tumors in female Sprague-Dawley rats. | Clomipramine. Dose: 25 mg/kg. Duration: 15 days | Between postnatal days 5 and 20, rat pups were injected daily with 25 mg/kg clomipramine, and handled either by holding them in a hand (H-handling) or by giving them a saline injection (I-handling), or left non-handled. During these manipulations, but not later, body weight gain was lower in the I-handled and clomipramine-treated pups than in the H-handled rats. | The percentage of animals developing mammary tumors was significantly higher, and the length of survival shorter among the clomipramine-treated rats than among the I-handled rats. However, both groups exhibited less tumors and longer survival than the non-handled controls. There were no differences in mammary tumor incidence or survival between the non-handled and H-handled rats. | Yes. Early postnatal treatment with clomipramine promoted DMBA-induced tumor growth in rats. This finding suggests that the increased depressive behavior exhibited by these animals may be associated with elevated incidence of mammary tumors and impaired survival. Further, clomipramine is commonly used in the treatment of depression in pregnant and nursing mothers. Recent clinical and experimental data suggest that early exposure to clomipramine has a number of hazardous effects on the developing organism. |
| Iishi (6) | 1993, Japan | The effects of the tricyclic antidepressant drug desipramine on the incidence, number and histology of colon tumors induced by azoxymethane (AOM), and on the serum norepinephrine (NE) concentration and the labeling index of colon mucosa were investigated in Wistar rats. | Desipramine. Dose & Duration: Rats were treated s.c. with 7.4 mg AOM/kg body wt once a week for 10 weeks, and also s.c. with 10 mg desipramine hydrochloride/kg body weight until the end of the experiment. |  | Treatment with desipramine significantly increased the incidence, but not the number, of colon tumors in week 35. However, it did not influence the location and the histological appearance of the colon tumors or the histological types of colon adenocarcinomas. | Yes. These findings indicate that desipramine enhanced the development of colon tumors and that its effect may be related to its effect in increasing proliferation of colon epithelial cells. |
| Jia (52) | 2008, China | To investigate the effects of mirtazapine and fluoxetine, representatives of the noradrenergic and specific serotonergic antidepressant (NaSSA) and selective serotonin reuptake inhibitor (SSRI) antidepressant respectively, on body weight, ingestive behavior, locomotor activity and tumor growth of human pancreatic carcinoma xenografts in nude mice. | Mirtazepine and fluoxetine. Dose: 10 mg/kg/d. Duration: 42 days | A subcutaneous xenograft model of human pancreatic cancer cell line SW1990 was established in nude mice. The tumor-bearing mice were randomly divided into mirtazapine group [10 mg/(kg/d)], fluoxetine group [10 mg/(kg/d)] and control group  (an equivalent normal saline solution) (7 mice in each group). Doses of all drugs were administered orally, once a day for 42 days. | There was no significant difference in tumor volume and tumor weight of the three groups. | No. Mirtazapine and fluoxetine have no effect on the growth of pancreatic tumor. Mirtazapine neither inhibits nor promotes pancreatic tumor growth according to the findings from this study. However, mirtazapine can significantly increase food intake and improve nutrition compared with fluoxetine in a pancreatic cancer mouse model. The results support the hypothesis that mirtazapine as an adjuvant therapy is superior to fluoxetine for pancreatic cancer patients with depression. |
| Kelvin (53) | 1989, UK | The oral toxicology of paroxetine has been evaluated in repeat-dose studies of up to 12 months’ duration in rats (Sprague DawIey) and rhesus monkeys, in a range of genetic toxicity studies, and in two-year carcinogenicity studies in rats and mice (outbred albino). | Paroxetine. Dose: 1-50 mg/kg/day (rats), 1-25 mg/kg/day (mice), and 0.8-6 mg/kg/day (monkeys). Duration: 12 months in rats and rhesus monkeys, and 2 years in rats and mice |  | *In vitro* studies comprised DNA repair in E. *coli* and unscheduled DNA synthesis in HeLa cells (genetic endpoint: repairable DNA damage), Ames’ tests in five strains of *S. typhimurium* (point mutation), mouse lymphoma assay (gene mutation), and cytogenetic assay in human lymphocytes (chromosomal damage). *In* *vivo* studies comprised the micronucleus test in mice and the dominant lethal test in male rats  (chromosomal damage). No genotoxic effects of paroxetine were observed in this battery of tests. | No. No genotoxic or carcinogenic activity has been found. In male mice, the incidence of malignant liver cell tumors was greater in groups given low and intermediate doses than in controls. This was significant only for the intermediate dose, and there were no significant dose-related trends. It is concluded that the increase in tumor incidence in intermediate-dose males was fortuitous. In rats, the incidence of lympho-reticular cell tumors in high-dose males was slightly increased compared with controls, but this did not reach statistical significance (P=0.06). There was a significant trend with dose (P<0.01); however, the overall incidence was low and within the laboratory background control range. It is concluded that the increase in tumors is of no toxicological significance. |
| Lee (74) | 2009, South Korea | Using a MTT reduction cell variability assay, fluoxetine-induced cell death in human ovarian cancer cell lines OVCR-3 and SK-OV-3 was examined. | Fluoxetine. Dose: 0-25mg. Duration: 1-24 hrs | Fluoxetine induced cell death in human ovarian carcinoma cell lines OVCR-3 and SK-OV-3 was established via a MTT cell viability assay. Fluoxetine induced cell death in a dose-dependent manner (SK-OV-3 cells were less sensitive to fluoxetine than OVCAR-3 cells). | Fluoxetine induced mitochondrial membrane permeability change and formation of reactive oxygen species leading to cell death. | No*. Fluoxetine may exhibit apoptotic effect against ovarian cancer sell lines by inducing the mitochondrial membrane permeability change. |
| Lin (75) | 2010, Canada | Investigated the mechanism of sertraline in downregulating protein synthesis that is associated with antiproliferative activity against breast cancer cells. | Sertraline. Cells:  Dose: 0-40 μmol/L Duration 20-24 hrs. Mice:  Dose: 20 mg/kg daily  Duration: 5 days | MCF-7 cells were incubated with 0-40 μmol/L of sertraline for 20-24 hrs. Mice were injected with doxorubicin (once at 10 mg/kg), rapamycin (4 mg/kg daily for 5 d), and sertraline (20 mg/kg daily for 5 d) following development of tumors from injected secondary lymphoma cells. | Sertraline was found to reduce cell growth, protein synthesis, and polysome distribution in the cells. Sertraline inhibits translation initiation by targeting assembly of the eIF4F complex and inducing phosphorylation of eIF2α. | No.* Sertraline causes significant antiproliferative activity. Combined sertraline and doxorubicin treatment in tumors induced an apoptotic response and decreased cell proliferation. |
| Merry (54) | 1991, UK | The authors investigated the effects of clomipramine on the development of pleiotropic drug resistance (PDR) in a solid tumor, the Ridgway osteogenic sarcoma (ROS and ROS/ADX/ G2). | Clomipramine. Dose: 10 μg/g. Duration: 24 – 48 h. |  | In all cases, PDR could be partly or completely overcome by treatment with non-cytotoxic doses of clomipramine. 2 treatments with 10 μg/g completely overcame resistance to actinomycin D and vincristine, | No*. Evidence suggests that PDR may contribute to treatment failure. PDR was partly or completely overcome by treatment with non-cytotoxic doses of clomipramine. |
| Rosetti (67) | 2006, Italy | The authors compared two SSRIs, one very specific for serotonin reuptake transporters (paroxetine) and another which also inhibit norepinephrine and dopamine transporters (venlafaxine), for their ability to counteract growth of various murine and human cancer cell lines. | Paroxetine and venlafaxine. *In vitro* dose: 0 – 100 μM in cultured cells’ growth media |  | The ability of paroxetine to counteract the growth of various types of cancer cells, in vitro, was investigated. Paroxetine has cytotoxic activity against tumor cells, both of murine or human origin in the micromolar concentration range. Venlafaxine exhibited a very weak cytotoxic effect on all the various cancer cell lines investigated. | No*.The presence of apoptosis induction after paroxetine treatment was confirmed. Paroxetine induced cell death both to primary and to long cultured cells. Micromolar concentrations of certain SSRIs can induce a cytotoxic effect on cancer cells, more marked that on non cancerous cells. Nevertheless, the applicability of SSRIs as anticancer agents appears limited by the fact that effective concentrations are much higher than those found in patients under standard antidepressant therapeutic regimens with the same drugs. |
| Sauter (55) | 1989, Switzer-land | The author evaluated the cytostatic activity of tricyclic antidepressants on human renal cancer cells *in vitro*. | Imipramine (IMI) and clomipramine (CLOM). *In vitro* dose: 30 – 180 μM in cultured cells’ growth media |  | IMI produced detectable tumor cell growth inhibition at 36 μM and complete growth inhibition at 180 μM; CLOM produced detectable tumor cell growth inhibition at 30 μM and complete growth inhibition at 80 μM | No*. Complete growth inhibition of cultured human renal cancer cells was observed with imipramine and clomipramine at concentrations similar to that of nitrogen mustard. |
| Serafeim (56) | 2003, UK | In a previous study the authors noted that at concentrations only slightly higher than those required for blocking serotonin-promoted apoptosis, the SSRIs began to exhibit what appeared to be direct inhibitory effects on biopsy-like BL cells maintained in early passage.20 This preliminary observation led us to explore possible proapoptotic activity of the SSRIs in their own right. | Fluoxetine, paroxetine, and citalopram | Burkitt lymphoma (BL) L3055 cells were treated for 24 hours with 0.5 to 30 μM fluoxetine; 0.5 to 30 μM paroxetine; and 1.0 to 150 μM citalopram. | 3 structurally distinct SSRIs—fluoxetine, paroxetine, and citalopram—act directly on Burkitt lymphoma (BL) cells to trigger rapid and extensive programmed cell death. Resultant SSRI-induced apoptosis was preceded by caspase activation, poly(ADP-ribose) polymerase-1 (PARP-1) cleavage, DNA fragmentation, a loss of mitochondrial membrane potential, and the externalization of phosphatidylserine, and reversed by the overexpression of *bcl*-2. | No*. Each of the 3 structurally distinct SSRIs investigated was found capable of driving cessation of DNA synthesis in group I BL cells remaining faithful to the original biopsy phenotype. |
| Shoyab  (57) | 1982, US | Assess the effects of tricyclic antidepressants on the binding of the tumor promoter [^3^H]PDBu to its specific receptors (murine BMF). | Imipramine (IMI) and clomipramine (CLOM) |  | The dose required for 50% inhibition of [^3^H]PDBu to murine BMF: IMI (3.5 x 10^-3^ M) and CLOM (9 x 10^-4^ M). CLOM was more potent than IMI in competing for [^3^H]PDBu receptor binding. | No. The competitive inhibition of [^3^H]PDBu binding by imipramine and clomipramine suggests that these drugs could act either as agonists or as antagonists of tumor-promoting diterpene esters. |
| Slamon (58) | 2001, UK | The effects of acute (24 h) exposure to antidepressants to DNA damage in cultured C6 rat glioma cells were determined. | Amitriptyline (AMI), imipramine (IMI), fluoxetine (FLX) and tranylcypromine (TCP) | *In vitro* exposure:  AMI/IMI: 3.2 to 320 μM; FLX: 2.9 to 290 μM; and TCP: 1.2 to 5.9 mM | There were increases in DNA damage with increasing concentrations of antidepressants. At the higher concentration of antidepressants the damage observed was indicative of apoptotic (extensive) damage or ablative damage. FLX exposure caused the greatest degree of DNA degradation. The concentrations at which the cytotoxic effects become apparent are comparable to therapeutic doses. | No*. Antidepressants induce significant amounts of DNA damage in C6 rat glioma cells. Intracellular DNA damage is coupled with fluoxetine cytotoxicity in C6 cells. DNA degradation may serve a protective function by limiting gene transfer in dying cells. |
| Sonier (59) | 2006, Canada | Certain studies suggest that some selective serotonin re-uptake inhibitors promote breast cancer in animals and humans. This study attempted to clarify the role of serotonin in promoting the growth of neoplastic mammary cells. | The effects of 5-HT and a specific 5-HT_2A_ receptor agonist, DOI (2,5-dimethoxy-4-iodoampheta-mine hydro-chloride), on the proliferation of MCF-7 cells were determined by MTT cell proliferation assays. | Expression of the 5-HT_2A_ serotonergic receptor subtype in MCF-7 cells was determined by RT-PCR, Western blotting, and immunofluorescence analysis. The mitogenic effect of 5-HT on MCF-7 cells was determined by means of the MTT proliferation assay. | The 5-HT_2A_ receptor subtype is fully expressed in the MCF-7 human breast cancer cell line, in terms of encoding mRNA and receptor protein. MTT proliferation assays revealed that 5-HT and DOI, a selective 5-HT_2A_ receptor subtype agonist, stimulated MCF-7 cell. Results also suggest that 5-HT initiates a mitogenic effect in MCF-7 cells and that this positive proliferative effect is mediated at least in part by the 5-HT_2A_ receptor subtype. | Yes. The results indicate that 5-HT plays a mitogenic role in neoplastic mammary cells. The data also indicate that 5-HT exerts this positive growth effect on MCF-7 cells through, in part, the 5-HT_2A_ receptor subtype, which is fully expressed in this cell line. |
| Spanova (72) | 1997, Czech Republic | Gel electrophoresis of DNA was used to estimate DNA changes in C6 rat glioma cells following treatment with psychotropic drugs. | Amitriptyline (AMI), imipramine (IMI) and fluoxetine (FLX) | In vitro exposure of C6 cells to AMI, IMI and FLX: final concentration of 1 x 10^-6^ M and/or 5 x 10^-6^ M for 1 to 5 days | The most pronounced DNA fragmentation was detected in C6 glioma cells treated with fluoxetine – the DNA breaks that occurred after fluoxetine (1.0 μM and 5.0 μM) exposure for 24 h were apoptosis-induced. | No*. Psychotropic drugs provoked DNA changes in C6 glioma cells – these changes were verified as apoptotic in cells treated with fluoxetine. |
| Stepulak (71) | 2008, Poland | The authors studied the effect of FLX on tumor growth in lung (A549), colon (HT29), neuroblastoma (SKNAS), medullo-blastoma/rhabdomyo-sarcoma (TE671), astrocytoma (MOGGCCM) and breast (T47D) cancer cells and explored potential mechanisms of its action. | Fluoxetine (FLX) | To determine the effect of fluoxetine on kinases activity, A549 cells were incubated with different concentrations (1–10 ìM) of FLX for indicated time periods. Cell proliferation was assessed after 48–96 hours by using the MTT method. | FLX reduced growth of cancer cells *in vitro* in a concentration dependent manner. The antiproliferative effect of FLX was already evident after 24 hours exposure and more pronounced at 96 hours. | No*. Fluoxetine reduces cancer cell growth in vitro through a mechanism most likely not related to serotonin reuptake inhibition. Inhibition of ERK1/2 kinases, followed by changes of gene expression of cell cycle regulating genes, represents one potential mechanism for the observed anticancer effect. |
| Toth (60) | 1975, US | Review of carcinogenic synthetic and naturally occurring hydrazines | 1-Isonicotinoyl-2-isopropyl-hydrazine (Iproniazid) |  | Many synthetic and naturally occurring hydrazines have displayed carcinogenicity | Yes |
| Tsuruo (61) | 1982, Japan | The authors evaluated the ability of clomipramine to potentiate the chemotherapeutic effect of vincristine in vincristine resistant tumor bearing mice. | Clomipramine. Dose: 20 to 50 mg/kg. Duration: 10 days |  | Although vincristine alone did not confer a significant therapeutic effect in vincristine resistant P388 leukemia-bearing mice, co-administration of clomipramine did enhance the chemotherapeutic efficacy of vincristine in these mice. The mechanism of the enhancement of vincristine cytotoxicity by clomipramine is the inhibition of vincristine efflux function of tumor cells, especially vincristine resistant tumor cells. | No*. Although the *in vivo* circumvention of vincristine resistance was not achieved perfectly, the authors speculated that more than 98-99% of vincristine resistant tumor cells which could not be killed by vincristine alone could be killed by the co-administration of clomipramine. |
| Tucker (62) | 1983, US | This brief overview summarizes the important details of the preclinical toxicological studies of bupropion. | Bupropion. Dose: from 50 to 300 mg/kg/day in mice and rats. |  | Bupropion did not cause an increase in tumors in rats or mice after lifetime administration, nor was the time to onset of spontaneous tumors affected. | No. No evidence of bupropion-related carcinogenicity. |
| Tutton & Barkla (63) | 1976, Australia | Epithelial cell proliferation was studied in the jejunum and in the colon of normal rats, in the colon of dimethylhydrazine-treated rats and in dimethylhydrazine-induced adeno-carcinoma of the colon using a stathmokinetic technique. Estimates of cell proliferation rates in these four tissues were then repeated in animals which had been depleted of biogenic amines by treatment with reserpine and in animals whose monoamine oxidase was inhibited by treatment with nialamide. | Nialamide | 13 rats were injected with nialamide (Niamid) at a dose of 100 mg/kg 4 h prior to the commencement of the estimate of metaphase rate. | In amine-depleted animals cell proliferation essentially ceased in all four tissues examined. Inhibition of monoamine oxidase did not significantly influence cell proliferation in nonmalignant tissues but accelerated cell division in colonic tumors. The observed difference between the responses of normal and of neoplastic cells to inhibition of monoamine oxidase could be due to a difference in the location of amine receptors in the two cell types. | Yes. Whatever the explanation of the above result the finding that tumor cell proliferation can be manipulated by drugs which are known to interact with biogenic amines may be significant from several aspects. Firstly, altered amine metabolism may be a feature of tumor biology which is fairly accessible to investigation and experiments in this field are currently being undertaken in this laboratory. Secondly, chemotherapy and radiotherapy for cancer patients may be improved by pharmacological manipulation of amine metabolism. |
| Tutton & Barkla (64) | 1982, Australia | In the present study, two specific inhibitors of serotonin uptake,  Citalopram and fluoxetine, are examined for their effects on cell proliferation and tumor growth. | Citalopram and fluoxetine | Xenograft-bearing mice received either twice-daily i.p. injections of citalopram (20 or 40 mg/kg) or once daily injections of fluoxetine (10 or 20 mg/kg). | Citalopram and fluoxetine were each found to suppress cell division in dimethylhydrazine-induced colonic tumors in rats, and to retard the growth of 2 out of 3 lines of human colonic tumors propagated as xenografts in immune-deprived mice. | No*. The preceding results clearly indicate that the growth of some, but not all, colonic tumors is retarded by the serotonin-uptake-inhibiting drugs citalopram and fluoxetine.  The response of colonic carcinomas to serotonin-uptake inhibitors lends direct support to the concept that colonic tumor cells, unlike jejunal or colonic crypt cells, have a serotonin-uptake mechanism. |
| Volpe (65) | 2003, US | Previous publications have suggested that commonly prescribed antidepressants have the potential to stimulate the proliferation of extant tumors in human and rodent in vivo and in vitro models. The direct effects of amitriptyline and fluoxetine were evaluated in assays that detect different aspects of proliferative responses at pharmacologically relevant drug concentrations. | Amitriptyline and fluoxetine | 3 *in vitro* assays of cellular proliferation and clonal growth were used with human (MCF7, PA-1 and LS174T) and murine (B16.f10, C-3 and B16.f1) tumor cell lines. The cells were exposed to amitriptyline or fluoxetine (0.001–100 ìM) for different time periods (1–7 days) and at varying serum concentrations (0.1–15%). | Amitriptyline and fluoxetine failed to significantly stimulate tumor cell proliferation, DNA synthesis, or colony formation. Both drugs inhibited B16.f10 colony growth at concentrations above 5 ìM along with significant suppression of DNA synthesis in B16.f10 and C-3 cells at 30 ìM. The results were consistent between the three assays in demonstrating either inhibitory or no activity with amitriptyline or fluoxetine. | No*. Amitriptyline and fluoxetine consistently demonstrated inhibitory or no activity with the human or murine tumor cell lines in conventional *in vitro* assays of cell proliferation and clonogenicity in optimal or suboptimal culture conditions. The results on the lack of tumor growth-promoting activity of amitriptyline and fluoxetine are contrary to the previous reports of *in vitro* stimulation, instead confirming the inhibitory results by other investigators for these two drugs. |
| Wright, (66) | 1994, US | Recent evidence supports the concept that tumor growth in vivo depends on evasion of normal homeostatic control mechanisms that operate through induction of cell death by apoptosis. This study tested the hypothesis that a common property shared by known or suspected tumor promoters is the ability to block the process of apoptosis. A total of 10 tumor promoters were tested and all were found to inhibit DNA fragmentation and cell death of 7 different cell lines triggered into apoptosis by diverse agents. | Amitriptyline and fluoxetine | U937 cells were cultured in the presence of different physiological concentrations of  Elavil for a total of 7 days with periodic testing for sensitivity to TNF-induced apoptosis. The cells gradually acquired resistance to apoptosis, with virtually 100% resistance expressed on day 7. The kinetics of this phenomenon were dose-related as cells cultured in the highest concentration of Elavil (0.4 ìM) acquired resistance more rapidly than cells cultured in the lowest concentration (0.1 ìM). | Resistance to apoptosis could be induced rapidly (within 1 h) by treating with relatively high concentrations of promoters. However, low physiological concentrations of promoters could also induce complete resistance to apoptosis after prolonged exposure (5-15 days of culture). Like tumor promotion in vivo, promoter-induced resistance to apoptosis was reversible after culturing in the absence of promoter**.** The antidepressants Elavil (amitriptyline HC1) and Prozac (fluoxetine HCI) are suspected tumor promoters based on a recent report that they augment tumor growth in animals. These drugs also inhibited DNA fragmentation, although at concentrations much higher than those achieved clinically in patients under treatment for depression. | Yes. Elavil (0.1 ìM) inhibited DNA fragmentation at a concentration even lower than serum levels (0.26-0.4 ìM) in patients receiving a typical dose of 100 mg/day of this antidepressant. These findings suggest that prolonged exposure to low physiological levels of tumor promoters will lead to the acquisition of resistance to apoptosis.  These findings provide new insight into the mechanism of tumor promotion and suggest a novel in vitro screening assay to detect new tumor-promoting agents in the environment. |
| Xia (69) | 1999, Sweden | Some widely used antidepressants such as imipramine, clomipramine, and citalopram have been found to possess antineoplastic effects. In the present study, these compounds were found to induce apoptotic cell death in human acute myeloid leukemia HL-60 cells. | Imipramine, clomipramine and citalopram. Dose: 80 ìM imipramine, 35 ìM clomipramine, or 220 ìM citalopram | Apoptosis was induced in human acute myeloid leukemia cell line HL-60 cells by 80 ìM imipramine, 35 ìM clomipramine, or 220 ìM citalopram.  For studies on the inhibition of apoptosis, the cells were preincubated for 1 hour with 20, 50, 100, or 200 ìM zVAD-fmk, prior to addition of the antidepressants. | Treatment with apoptosis-inducing concentrations of antidepressants (80 ìM imipramine, 35 ìM clomipramine, or 220 ìM citalopram) caused induction of caspase-3/caspase-3-like activity, which was monitored by the cleavage of poly(ADP-ribose) polymerase (PARP), the loss of the 32 kD caspase-3 (CPP32) precursor, and the cleavage of the fluorescent CPP32-like substrate PhiPhiLux. Pretreatment with a potent caspase inhibitor benzyloxy-carbonyl-Val-Ala-Asp-fluoro-methyl-ketone (zVADfmk) inhibited antidepressant-induced CPP32/CPP32-like activity and apoptosis. | No*. These results suggest that these antidepressants may induce apoptosis via a caspase-3-dependent pathway, and induction of apoptosis by these antidepressants may provide a clue for the mechanism of their antineoplastic effects. |

* Indicates that antiproliferative and/or antineoplastic properties (e.g., cytotoxicity, genotoxicity, apoptosis, DNA fragmentation/degradation, etc.) were identified in one or more of the tested antidepressant
