# Supplementary material for: Antidepressants and Breast and Ovarian Cancer Risk: A Review of the Literature and Researchers' Financial Associations with Industry
Source: PLoS One. 2011 Apr 6;6(4):e18210. doi: 10.1371/journal.pone.0018210 (PMC3071810; doi:10.1371/journal.pone.0018210)
Supplement: Table S4 — Results regarding carcinogenicity from pre-clinical studies. (DOC) [file pone.0018210.s005.doc]

Table S4  Results regarding carcinogenicity from pre-clinical studies

| **Study Design** | **Yes** | **No/Antiproliferative*** | **Total** |
| --- | --- | --- | --- |
| Animal | 7 | 17 / 12* | 24 |
| Lab | 3 | 8 / 8* | 11 |
| Total | 10 | 25 / 20* | 35 |

*A total of 20 studies reported that ADs not only did not have carcinogenic properties but were reported to have antiproliferative properties.
